# 2,3,5,4′-Tetrahydroxystilbene-2-*O*-β-D-Glucoside improves female ovarian aging

**DOI:** 10.3389/fcell.2022.862045

**Published:** 2022-08-30

**Authors:** Hung-Yun Lin, Yung-Ning Yang, Yi-Fong Chen, Tung-Yung Huang, Dana R. Crawford, Hui-Yu Chuang, Yu-Tang Chin, Hung-Ru Chu, Zi-Lin Li, Ya-Jung Shih, Yi-Ru Chen, Yu-Chen S. H. Yang, Yih Ho, Paul J. Davis, Jacqueline Whang-Peng, Kuan Wang

**Affiliations:** ^1^ Graduate Institute of Cancer Biology and Drug Discovery, College of Medical Science and Technology, Taipei Medical University, Taipei, Taiwan; ^2^ TMU Research Center of Cancer Translational Medicine, Taipei Medical University, Taipei, Taiwan; ^3^ Traditional Herbal Medicine Research Center of Taipei Medical University Hospital, Taipei Medical University, Taipei, Taiwan; ^4^ Pharmaceutical Research Institute, Albany College of Pharmacy and Health Sciences, Albany, NY, United States; ^5^ Cancer Center, Wan Fang Hospital, Taipei Medical University, Taipei, Taiwan; ^6^ School of Medicine, I-Shou University, Kaohsiung, Taiwan; ^7^ Department of Pediatrics, E-DA Hospital, Kaohsiung, Taiwan; ^8^ Department of Immunology and Microbial Disease, Albany Medical College, Albany, NY, United States; ^9^ Graduate Institute of Clinical Medicine, College of Medicine, Kaohsiung Medical University, Kaohsiung, Taiwan; ^10^ Department of Obstetrics and Gynecology, Kaohsiung Medical University Hospital, Kaohsiung, Taiwan; ^11^ School of Dentistry, College of Oral Medicine, Taipei Medical University, Taipei, Taiwan; ^12^ Graduate Institute of Nanomedicine and Medical Engineering, College of Medical Engineering, Taipei Medical University, Taipei, Taiwan; ^13^ Joint Biobank, Office of Human Research, Taipei Medical University, Taipei, Taiwan; ^14^ School of Pharmacy, Taipei Medical University, Taipei, Taiwan; ^15^ Department of Medicine, Albany Medical College, Albany, NY, United States

**Keywords:** Polygoni multiflori extract, THSG, aging, fertility, aging-induced infertility

## Abstract

Reduced fertility associated with normal aging may reflect the over-maturity of oocytes. It is increasingly important to reduce aging-induced infertility since recent trends show people marrying at later ages. 2,3,5,4′-Tetrahydroxystilbene-2-*O*-β-D-glucoside (THSG), a polyphenol extracted from *Polygonum multiflorum*, has been reported to have anti-inflammatory and anti-aging properties. To evaluate whether THSG can reduce aging-related ovarian damage in a female mouse model of aging, THSG was administered by gavage at a dose of 10 mg/kg twice weekly, starting at 4 weeks of age in a group of young mice. In addition, the effect of THSG in a group of aged mice was also studied in mice starting at 24 weeks of age. The number of oocytes in the THSG-fed group was higher than in the untreated control group. Although the percentage of secondary polar bodies (PB2) decreased during aging in the THSG-fed group, it decreased much more slowly than in the age-matched control group. THSG administration increased the quality of ovaries in young mice becoming aged. Western blotting analyses also indicated that CYP19, PR-B, and ER-β expressions were significantly increased in 36-week-old mice. THSG also increased oocyte numbers in aged mice compared to mice without THSG fed. Studies of qPCR and immunohistochemistry (IHC) analyses of ovaries in the aged mice groups were conducted. THSG increased gene expression of anti-Müllerian hormone (AMH), a biomarker of oocyte number, and protein accumulation in 40-week-old mice. THSG increased the expression of pgc1α and atp6, mitochondrial biogenesis-related genes, and their protein expression. THSG also attenuated the fading rate of CYP11a and CYP19 associated with sex hormone synthesis. And THSG maintains a high level of ER-β expression, thereby enhancing the sensitivity of estrogen. Our findings indicated that THSG increased or extended gene expression involved in ovarian maintenance and rejuvenation in young and aged mice. On the other hand, THSG treatments significantly maintained oocyte quantity and quality in both groups of young and aged mice compared to each age-matched control group. In conclusion, THSG can delay aging-related menopause, and the antioxidant properties of THSG may make it suitable for preventing aging-induced infertility.

## Graphical Abstract

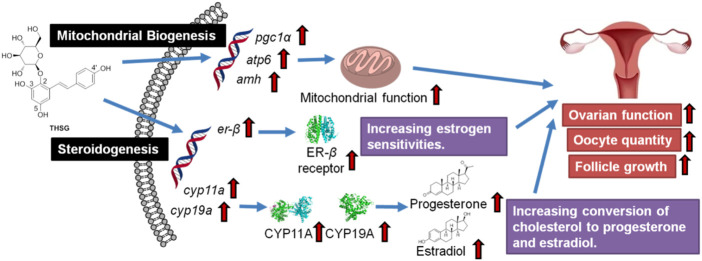

## Highlights

- Aging control mice had reduced ovarian reserve, leading to premature ovarian failure, which could be prevented by the administration of THSG.
- THSG treatment increased the expression of mitochondrial biogenesis-related genes and steroidogenesis-related genes, which were significantly activated and higher than the control group.
- THSG treatment maintained oocyte quantity and quality in young and aged mice.

## Introduction

The mammalian ovarian reserve consists of a limited number of primordial follicles that represent a female’s lifetime reproductive capacity (Grive and Freiman, 2015). In most mammals, ovarian reserve is created during embryonic and early postnatal development (Richardson et al., 2014). Human ovaries have oocyte numbers peaking in mid-to-late gestation and then undergoing a sharp decline that persists shortly after birth. The complex and diverse mechanisms of the follicle formation process are critical to achieving the perfect balance to ensure long-term fertility and reproductive health. The ovary is the earliest organ to age. Ovarian aging is like the pacemaker of aging in the female body and drives the aging of multiple organs in the body. Ovarian aging is characterized by a decline of follicles and oocytes in ovarian reserve, both in quantity and quality (Broekmans et al., 2009). Decreasing oocyte quality causes infertility in women. Reducing the size of the pool of oocytes causes a decrease in the secretion of inhibin B by the small follicles of the anterior antrum. The loss of inhibin increases the secretion of pituitary follicle-stimulating hormone (FSH) (Gregory and Kaiser, 2004). As FSH increases in the early stage of the follicle, aging ovaries show faster follicle development and earlier dominant follicle selection (Klein et al., 1996; de Koning et al., 2000). With age, the abundance of preterm follicles and the reduction of antral follicles first lead to a gradual increase in FSH levels, followed by the follicular phase of the irregular menstrual cycle, however, only after significant aging of the ovaries does this become apparent (den Tonkelaar et al., 1998; Klein et al., 2002; Broekmans et al., 2009). Endocrine disorders lead to ovulatory dysfunction, resulting in female infertility (McTavish et al., 2007; Gleicher et al., 2010).

Aging reproduction and subsequent ovarian dysfunction increase the prevalence of infertility (Fritz and Jindal, 2018). In addition, demographic and socioeconomic factors influence women in their mid-thirties to delay childbearing (Mihalas et al., 2017). The chances of giving birth reduce and the risk of aneuploidy, miscarriage, and birth defects significantly increase. Therefore, anti-ovarian aging has become a research topic of widespread concern for biomedical scientists and the pharmaceutical industry. In the past few years, significant progress has been made in exploring possible anti-ovarian drugs or approaches, such as calorie restriction mimetics, antioxidants, and inducers of autophagy. The causes of aging infertility are complicated. However, oxidative stress and quality of age-related oocyte deterioration (Mihalas et al., 2017), as well as the endocrine changes (Broekmans et al., 2009), are now well recognized. The main cause of age-related fertility decline is the accumulation of spontaneous mitochondrial damage caused by increased reactive oxygen species (ROS) in oocytes (Lim and Luderer, 2011; Elizur et al., 2014; Takeo et al., 2017). The increased ROS is generated by mitochondria themselves during their daily biological metabolism (Sasaki et al., 2019). Mitochondrial dysfunction reduces ATP synthesis and affects meiotic spindle assembly responsible for chromosome segregation. Furthermore, regeneration of aged oocytes reduces the reliability of protective mechanisms against ROS scavenging metabolism, repair of ROS-damaged DNA, and proteasome and autophagy systems against ROS-damaged proteins (Chaube et al., 2005; Choi et al., 2007; Tamura et al., 2008; Miyamoto et al., 2010; Liu et al., 2013). Thus, increased ROS and increased oocyte sensitivity to ROS lead to spindle instability, chromosomal abnormalities, shortened telomeres, and reduced developmental capacity of senescent oocytes (Wiener-Megnazi et al., 2004; Das et al., 2006; Tamura et al., 2008; Agarwal et al., 2012; Nuñez-Calonge et al., 2016; Sasaki et al., 2019).

In addition to mitochondrial ROS, multiple mitochondrial dysfunctional signaling pathways have been implicated in cellular senescence, with G1 cell cycle arrest in an irreversible state and insensitivity to growth factor stimulation. These include impaired mitochondrial dynamics involved in fusion and fission (Lee et al., 2007; Seo et al., 2010), defects in the mitochondrial electron transport chain (ETC) (Stöckl et al., 2007; Moiseeva et al., 2009), bioenergetic imbalance (Wang et al., 2003; Zwerschke et al., 2003; Stöckl et al., 2007), altered mitochondrial metabolism (Jiang et al., 2013; Salminen et al., 2014), and altered mitochondrial membrane potential (Wiel et al., 2014). These pathways highlight insights into understanding the impact of mitochondrial stress on aging, linking mitochondrial dysfunction and through the cellular aging process. However, the mechanisms by which these factors promote cellular senescence, and whether these pathways are conserved in all senescent cells, such as ovarian somatic and germ cells, are unclear.

Human bodies have developed different mechanisms to combat oxidative stress. Those mechanisms are mainly prevention, repair, and antioxidant mechanisms (Walczak-Jedrzejowska et al., 2013). In addition to inhibitors of NADPH oxidase and lipoxygenase, cofactors of antioxidant enzymes, as well as metal chelators, can counteract ROS levels that can be referred to as antioxidants (Roy and Nath, 2021). However, many of these antioxidants may function as a prooxidant due to the inherited redox potential of these substances in an appropriate environment (Gurer-Orhan and Suzen, 2015; Eghbaliferiz and Iranshahi, 2016). Endogenously enzymatic antioxidants such as superoxide dismutase enzymes (SODs), glutathione peroxidase, catalases, and glutathione oxidase are more efficacious as a free radical scavenging role. These antioxidants have transition metal cores which are capable of transfer of electrons essential for redox reaction with ROS and thereby, effectively neutralizing the adverse effects of ROS. Apart from these endogenous enzymatic antioxidants, thioredoxin plays a central role in humans as a defensive response to ROS facilitating the reduction of other proteins by cysteine thiol-disulfide exchange and playing an important role in cell survival (Fujii et al., 2005). In addition to endogenous antioxidants, studies have recognized the benefits of supplementary antioxidants to reduce ROS-induced damages. Myo-inositol is used to decrease levels of androgen and increase sensitivity to insulin improves ovarian function (Nestler, 1998). PUFAs promote the synthesis of prostaglandin and steroid hormone apart from contributing to the formation of cell membranes of the sperm and oocyte essential for fertilization (Wathes et al., 2007).

Clinically, *in vitro* fertilization (IVF) using donor oocytes may be the last resort in the treatment of age-related infertility. ROS may arise from cumulus cells, leucocytes, and culture media in assisted reproductive technology. Therefore, IVF cycles will yield better results if patients are screened for oxidative stress levels (Bedaiwy et al., 2004). Culture media are supplemented with antioxidants like β-mercaptoethanol, protein, vitamin E, vitamin C, cysteamine, cysteine, taurine and hypotaurine, and thiols to enhance the growth and maturation of embryos by downregulating the effects of ROS to scavenge ROS by antioxidants. Subsequently, it exerts favorable effects to reduce blastocyst degeneration, embryo apoptosis, and increase hatching of blastocysts (Agarwal et al., 2006). However, clinical studies report that IVF increases the risk of birth defects compared to natural conception (Zhao et al., 2020). Herein, developing some non-invasion agents for improving ovarian function and oocyte quality is ongoing. Several antioxidants, including vitamins D and E, *N*-acetylcysteine (NAC), resveratrol (3,5,4′-trihydroxy-*trans*-stilbene), coenzyme Q10, melatonin, folic acid, may improve infertility (Rodríguez-Varela and Labarta, 2020; Tesarik, 2021).

**FIGURE 1:**
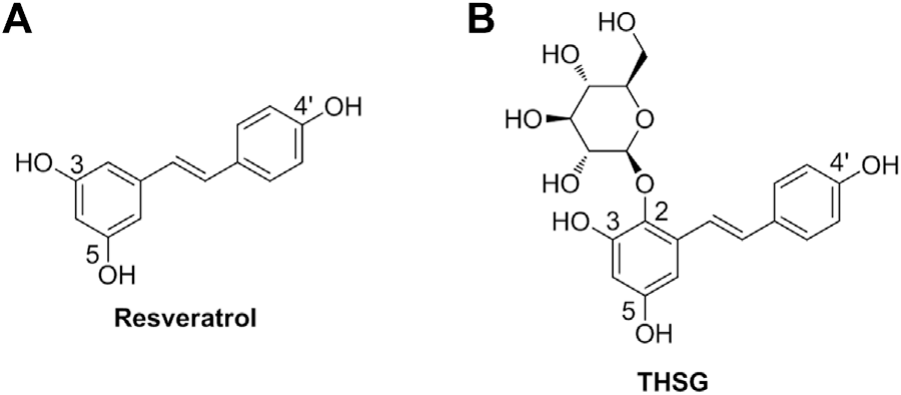
**(A)** The chemical structure of resveratrol (3,5,4′-trihydroxy-*trans*-stilbene). **(B)** The chemical structure of resveratrol glycosylated derivative (2,3,5,4′-tetrahydroxystilbene-2-*O*-β-D-glucoside; THSG).

Since their anti-inflammatory properties, naturally occurring polyphenols can modulate the host inflammatory response when admitted to animals. Certain flavonoids attract more attention based on their beneficial profiles in clinics (Shahidi and Ambigaipalan, 2015; Zhang et al., 2021). Resveratrol (Figure 1A) is a stilbene-based scaffold polyphenol found in a wide variety of natural plants (Pecyna et al., 2020). Resveratrol and its glycosylated derivative, 2,3,5,4′-tetrahydroxystilbene-2-*O*-β-D-glucoside (THSG) (Figure 1B) exhibit antioxidant and anti-inflammatory activities (Zhang et al., 2013b; Huang et al., 2013). Resveratrol activates antioxidant enzymes (Bhatt et al., 2011; Soufi et al., 2012) and the nuclear factor E2-related factor (Nrf2) antioxidant defense pathway (Wang et al., 2012). Resveratrol has been shown to prevent lipid peroxidation, especially low-density lipoprotein (LDL) lipid peroxidation (Brito et al., 2002). It can reduce oxygen and nitrogen free radicals to prevent the development of colitis in an animal model (Wang et al., 2008). The protecting effects of THSG are facilitated by the regulation of JNK, sirtuin1 (SIRT1), and NF-κB signal transduction pathways (Wang et al., 2009). Some studies have indicated that resveratrol and THSG stimulate SIRT1 activation (Ma et al., 2017). Recent studies revealed that resveratrol promoted oocyte maturation by improving mitochondrial function and SIRT1 activation in bovine (Wang et al., 2014), mice, and humans (Liu et al., 2018). Another study has also shown that resveratrol can improve the quality of postovulatory aging (POA) oocytes (Sun et al., 2019). In addition, a review article summarized that THSG can delay senescence and treat aging-related cardiovascular and neurological diseases (Ling and Xu, 2016). These results encouraged us to investigate whether THSG could protect mitochondria from superoxide damage and improve ovarian function.

In the current study, we examined the effects of THSG on fertility in young and aged mouse models. We proposed that the anti-oxidation properties of THSG can prevent oocytes from age-related injury in the aging and aged process. Studies aimed to investigate if THSG impacted mitochondrial biogenesis and the endocrine hormone-related factors to reduce aging damage in oocytes. Here we have demonstrated that THSG promoted the gene expression of mitochondrial function and steroidogenic activities, focusing on the oocyte numbers reserved in the ovarian.

## Materials and methods

### Preparation of Tetrahydroxystilbene-2-*O*-β-D-Glucoside from *Polygoni multiflori* extract

The extract of *Polygonum multiflorum* (PME) was obtained by immersing and refluxing the rhizomes of *P. multiflorum* in 50% ethanol at 65°C for 2 h. The filtrates were combined and concentrated to remove ethanol under the vacuum in a rotary evaporator. The extract was then lyophilized to powder. The purity of 2,3,5,4′-tetrahydroxystilbene-2-*O*-β-D-glucoside (THSG) was determined by HPLC analysis according to the method described previously (Chin et al., 2016). THSG (95% purity) was freshly dissolved in an ethanolic solution for animal study.

### Animal studies

**FIGURE 2:**
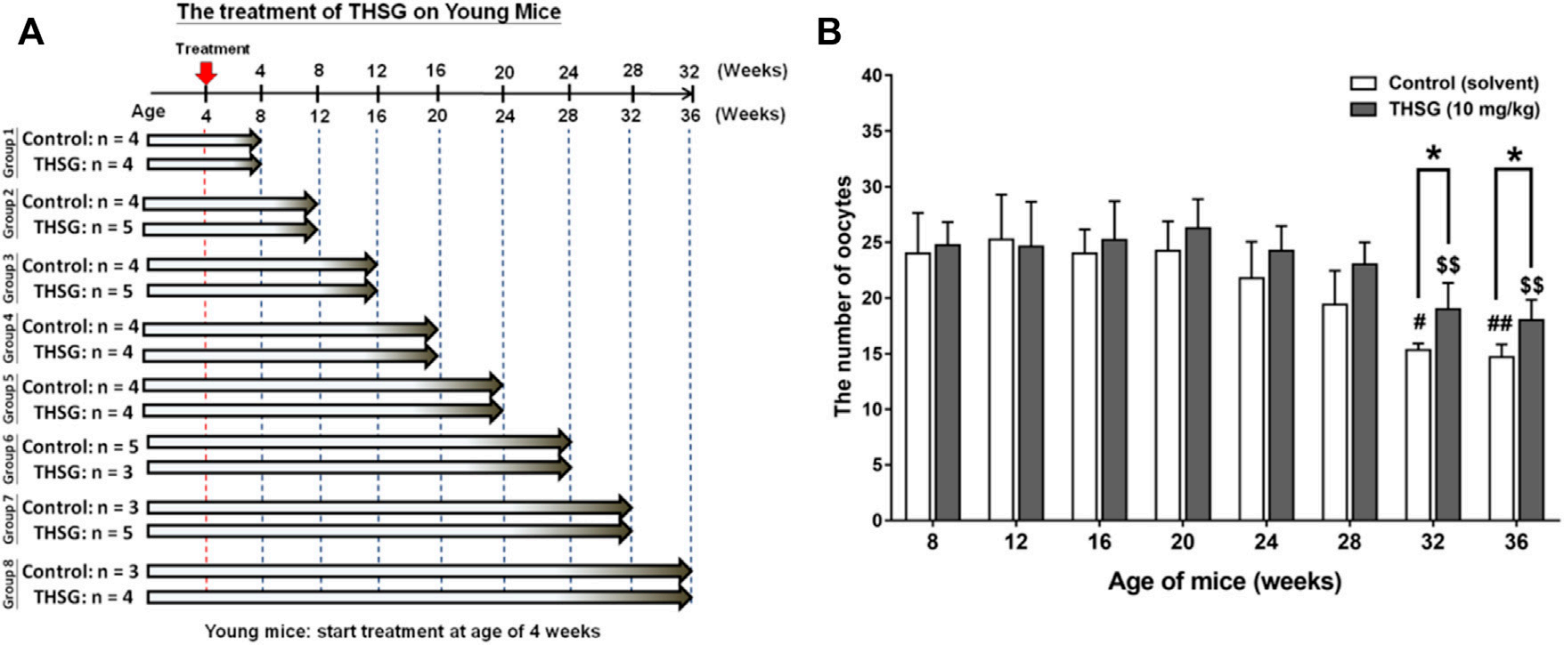
THSG treatment enhances oocyte quantity in young mice groups. **(A)** Female C57BL/6J mice (4 weeks-age) were received 10 mg/kg THSG (in 50% ethanol; experiment group) or solvent (50% ethanol; control group) for 32 weeks *via* gavage feeding. **(B)** Oocytes of each mouse were collected and counted from oviducts 14 h after hCG administration. Data are expressed as the mean ± SD; *p < 0.05, compared with each age-matched control group; #*p* < 0.05, ##*p* < 0.01, compared with the control group of 8-week-old mice; $$ *p* < 0.01, compared with the THSG-treated group of 8-week-old mice.

All animals used in this study were purchased from the National Laboratory Animal Center (NLAC) in Taipei, Taiwan, and the protocol was approved by the Animal Research Committee of TMU (IACUC-15-340). Four weeks old female C57BL/6J mice were housed in the animal center of Taipei Medical University under controlled environmental conditions (25°C, 12-h light/dark cycle, food and water were provided *ad libitum*). Mice were fed 10 mg/kg THSG in 50% ethanol (experiment group) or 50% ethanol (control group) twice weekly by gavage. After feeding for the period of 32 weeks (4- to 36-week-old mice represented the young mice groups) or feeding for the period of 16 weeks (24- to 40-week-old mice represented the aged mice groups), three to nine mice of control and experiment group were sacrificed every 4 weeks and collected ovary and oocytes for further studies. The precise numbers per group are illustrated in the flow diagram (Figures 2A, 5A).

### Collection of ovaries

Ovaries were randomly collected from each group (treated and untreated group of aging and aged mice) and immersion-fixed in 4% paraformaldehyde (Sigma-Aldrich) for 24 h. Tissue specimens were embedded in paraffin wax. The serial sections from each ovary were aligned in order on glass microscope slides by 5 μm thickness and stained with hematoxylin and eosin Y (HE, Sigma-Aldrich) and analyzed for morphology under light microscopy at different developmental stages (Liu et al., 2013). The follicles were categorized as primordial, preantral, antral, and preovulation according to the previous description (Myers et al., 2004). Follicles containing an oocyte surrounded by a single layer of squamous were classified as the primordial stage. Preantral follicles were identified as having more than one layer of granulosa cells with no visible antrum. Antral follicles contained an oocyte with a visible nucleus and possessed some small antral space. Mature antral follicles have a large antral space and are ready to release oocytes. Atretic follicles with a condensed oocyte or fragmented but still stained brightly with eosin Y. In the atretic stage, the zona pellucida remnants (ZPRs) were presented.

### Collection of oocytes

Control and experiment group of mice were induced superovulation by exogenous gonadotropin treatment—intraperitoneal injection with 5 IU pregnant mare’s serum gonadotropin (PMSG, Sigma, United States) for 48 h and then with 5 IU human chorionic gonadotropin (hCG, Sigma) 18 h before sacrifice. The ovaries were excised and washed twice in 37°C saline. Cumulus oocyte complexes (COCs) were physically retrieved from antral follicles in a HEPES-buffered tissue culture medium using a pair of 27-G needles under a stereomicroscope (Nikon, SMZ645, Tokyo, Japan) (Liu and Keefe, 2002). After washing 3-times with tissue culture medium, oocytes were then collected from young mice groups (8- to 36-week-old mice) and aged mice groups (28- to 40-week-old mice), only the MII oocytes that have extruded were collected in DMEM/F12 medium containing 10% fetal bovine serum (Eini et al., 2019).

### Western blotting analyses

Western blot analyses were conducted as previously described (Chen et al., 2022). The ovaries from 28 to 36 weeks aged-old female mice were harvested in the young mice groups. Total proteins were extracted and quantified. Protein samples were resolved by 10% sodium dodecylsulfate-polyacrylamide gel electrophoresis (SDS-PAGE). A 30-μg quantity of protein was loaded in each well with 5 µl sample buffer, and samples were resolved with electrophoresis at 100 V for 2 h. The resolved proteins were transferred from the polyacrylamide gel to Millipore Immobilon-PSQ Transfer polyvinylidene difluoride (PVDF) membranes (Millipore, Billerica, MA, United States) with Mini Trans-Blot^®^ Cell (Bio-Rad Laboratories). Membranes were blocked with a solution of 5% skim milk in Tris-buffered saline. Membranes were incubated with primary antibodies to CYP19, and ER-β (Santa Cruz), PR-B (Abcam), and GAPDH (GeneTex International, Hsinchu City, Taiwan) overnight at 4°C. Proteins were detected with horseradish peroxidase (HRP)-conjugated secondary antibodies and Immobilon™ Western HRP Substrate Luminol Reagent (Millipore). Western blots were visualized and recorded with the Amersham Imager 600 system (GE Healthcare Life Sciences, Pittsburgh, PA, United States). The densitometric analysis of Western blots was conducted with ImageJ 1.47 software (National Institute of Health, Bethesda, MD, United States) according to the software’s instructions.

### Immunohistochemistry

To determine the cellular localization of steroidogenic enzymes and related receptors in the ovary, studies of immunohistochemistry (IHC) were performed as described previously (Weenen et al., 2004; Valeri et al., 2020). Mouse ovaries were randomly collected from 24-, 28-, and 40-week-old female mice (*n* = 3) in the aged mice groups. In brief, ovarian tissue was embedded by paraffin. Sections of paraffin-embedded ovaries (5 μm) were collected on glass slides, then deparaffinized and hydrated. Antigen retrieval was achieved by microwaving the slides in 0.01 M sodium citrate solution (pH 6.0) for 15 min, cooling to room temperature, and washing three times in phosphate-buffered saline (PBS) each for 5 min. After washing, the slides were blocked with 3% hydrogen peroxide in PBS for 10 min to quench endogenous peroxidase activity. The nonspecific background was eliminated by blocking with 10% lamb serum in PBS for 1 h at room temperature. The slides were then incubated with the primary antibody at 4°C overnight. The primary antibodies included Anti-AMH antibody (ab103233, Abcam, Cambridge, United Kindom), Anti-CYP19 Antibody ((E-9) sc-374176, Santa Cruz, Dallas, Texas, US), and Anti-Estrogen Receptor-β antibody [(B-3) sc-373853, Santa Cruz] (1:100 dilution). The next day, after washing with PBS, the slides were incubated for 1 h at room temperature with biotinylated rabbit anti-mouse IgG or goat anti-rabbit IgG secondary antibody (1:200 dilution, Invitrogen, United States). The antibody dilution buffer was 10% lamb serum in PBS. Immunoreactive signals were detected using streptavidin-HRP and VECTOR Nova RED Peroxidase (HRP) Substrate Kit (Vectorlabs, Burlingame, CA, United States) at room temperature. Slides were counterstained with hematoxylin. A negative control was performed without the primary antibody incubation step. Immunostaining was observed using a Nikon Eclipse 50i microscope (Nikon, Tokyo, Japan) and captured by NIS Element F Software. Every antibody was repeated in slides from three separate ovaries with identical genotypes.

### Quantitative reverse transcriptase PCR

To examine the gene expressions, total RNA was extracted from whole ovaries (*N* = 10) by illustra™ RNAspin Isolation Kits, (GE Healthcare, United States). 1 μg of DNase I-treated total RNA was reversed transcribed with RevertAid H Minus Reverse Transcriptase (Life Technologies) into cDNA, and used as the template for real-time PCR reactions and analysis. Real-time PCR reactions were performed by using iQ™ SYBR^®^ Green Supermix PCR Kit on CFX Connect Real-Time PCR system (Bio-Rad) as described previously (Chin et al., 2018). As stated in the manufacturer’s instructions, this includes 5 min of initial denaturation at 95°C, followed by 40 rounds of denaturation at 95°C for 5 s, and 10 s of annealing/elongation at 60°C for 10 s as described previously (Ho et al., 2020). The primer sequences were shown as follows: *pgc-1α*, accession number NM_031347, forward (5′-GAA​TCA​AGC​CAC​TAC​AGA​CAC​CG-3′) and reverse (5′-CAT​CCC​TCT​TGA​GCC​TTT​CGT​G-3′); *atp6*, accession number MN400556.1, forward (5′-AGC​TCA​CTT​GCC​CAC​TTC​CT-3′) and reverse (5′-AAG​CCG​GAC​TGC​TAA​TGC​CA-3′); *amh*, accession number NM_000479.5, forward (5′-GGG​GCA​CAC​AGA​ACC​TCT-3′) and reverse (5′-GCA​CCT​TCT​CTG​CTT​GGT​TG-3′); *cyp11a*, accession number NM_000781.3, forward (5′-TTG​GTT​CCA​CTC​CTC​AAA​GC-3′) and reverse (5′-CCA​AAG​TCT​TGG​CTG​GAA​TC-3′); *cyp19a*, accession number NM_000103.3, forward (5′-GAG​CAT​GTT​AGA​GGT​GTC​CAG​CA-3′) and reverse (5′-GAC​TCT​CAT​GAA​TTC​TCC​ATA​CAT​CT-3′); *er-β*, accession number BC024181.2, forward (5′-TTC​TTT​CTC​ATG​TCA​GGC​ACA-3′) and reverse (5′-CTC​GAA​GCG​TGT​GAG​CAT​T-3′; *GAPDH*, accession number NM_002046.7, forward (5′-CAT​CAC​TGC​CAC​CCA​GAA​GAC​TG-3′) and reverse (5′-ATG​CCA​GTG​AGC​TTC​CCG​TTC​AG-3′).

### Statistical analysis

Data are presented as the mean ± SD. The data were analyzed using IBM^®^SPSS^®^ Statistics software, version 20.0 (SPSS Inc., Chicago, IL, United States). Student’s *t*-test was conducted, and changes were considered significant at *p* < 0.05 (*, #, $), *p* < 0.01 (**, ##, $$) and *p* < 0.001 (***, ###, $$$).

## Results

### Tetrahydroxystilbene-2-*O*-β-D-Glucoside treatment defers the degradation of oocyte quantity and quality in young mice groups

Starting at age 4-weeks old, young mice were fed THSG 10 mg/kg twice a week by oral gavage for different periods (Figure 2A). Mice fed with or without THSG were sacrificed at the end of every 4 weeks. This process continued until the end of the entire treatment period of 32 weeks. Ovaries were harvested. Oocytes were collected and counted. The number of oocytes collected from 24-week-old mice fed without THSG began to reduce slightly, although the difference between the control and THSG groups was not significant (Figure 2B). The 32-week-old mice in the THSG-untreated group had significantly fewer oocytes compared to the 24-week-old mice. However, in the THSG-fed group of 32-week-old mice, the number of oocytes was significantly increased. Mice administered with THSG were better at maintaining oocytes than age-matched controls. More pronounced differences were observed in 32- and 36-week-old mice, compared to younger mice 28 weeks earlier. The number of oocytes in the THSG-fed group was higher than in the untreated control group. Although the percentage of secondary polar body (PB2) dropped in the THSG-fed group during aging, its decreasing speed was far slower than in the age-matched control (Supplementary Figure S1). These results suggested that THSG can delay aging-related infertility in the young mice groups.

### Tetrahydroxystilbene-2-*O*-β-D-Glucoside increases preantral and antral follicles as well as activation of oocytes in young mice groups

**FIGURE 3:**
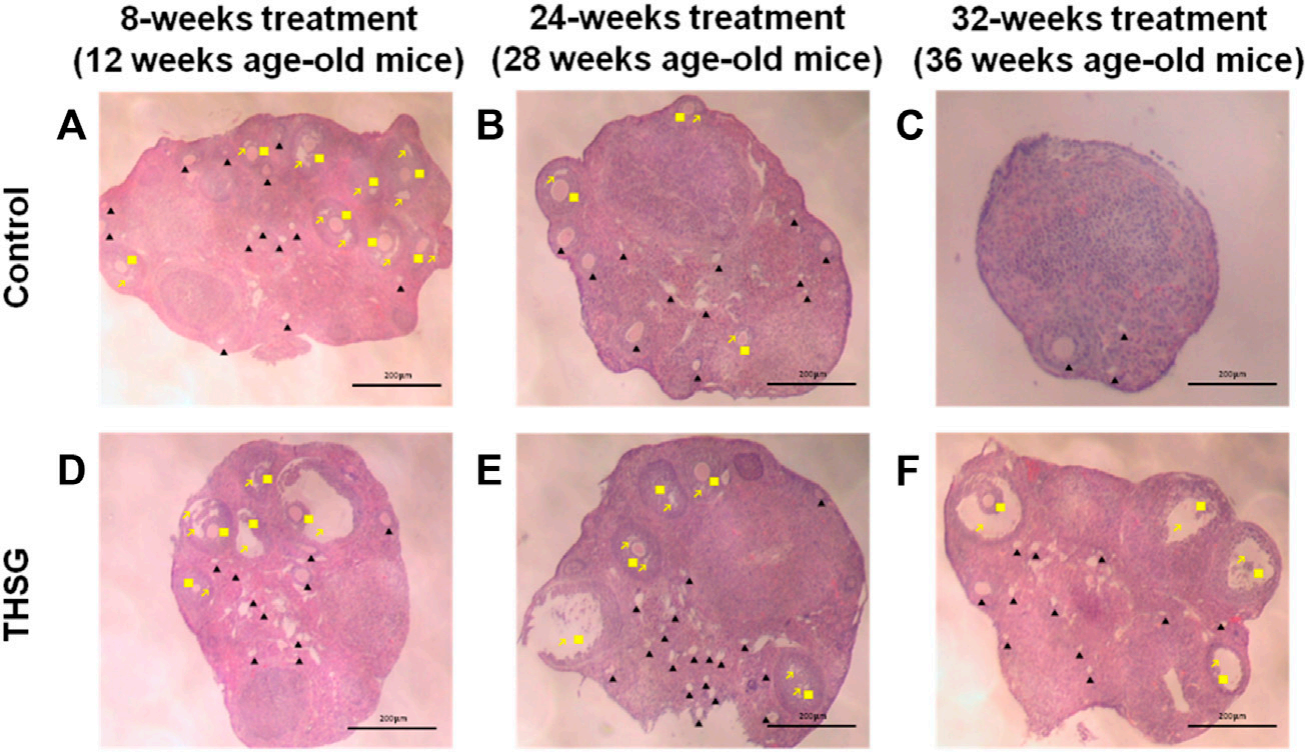
THSG increases preantral and antral follicles in aging young mice. The ovaries of 8-weeks, 24-weeks, and 32-weeks mice fed with or without THSG were collected and performed hematoxylin and eosin stain in control groups **(A–C)** and THSG-fed groups **(D–F)**. Different follicular stages were observed: primordial and preantral follicle (black triangle), antral follicle (yellow square) and antral spaces (yellow arrowhead).

Ovaries from young mice (12-weeks-old mice) showed large numbers of primordial and preantral follicles, accompanied by mature follicles (antral phase of follicles) (Figures 3A,D). Aged mice not fed THSG exhibited significantly reduced numbers of preantral and antral follicles, in particular, no mature (antral) follicles were observed. (Figure 3C). However, the number of primordial and preantral follicles increased in mice with THSG administration (Figures 3E,F) compared to the age-matched controls (Figures 3B,C). After 32 weeks of THSG treatment (36-week-old mice), there is more obvious follicle growth and the enlargement of antral spaces (Figure 3F) than in control (Figure 3C). There were numerous mature and growing follicles located in the ovarian cortex. These results suggest THSG can defer oocyte degradation and keep their quality.

**FIGURE 4:**
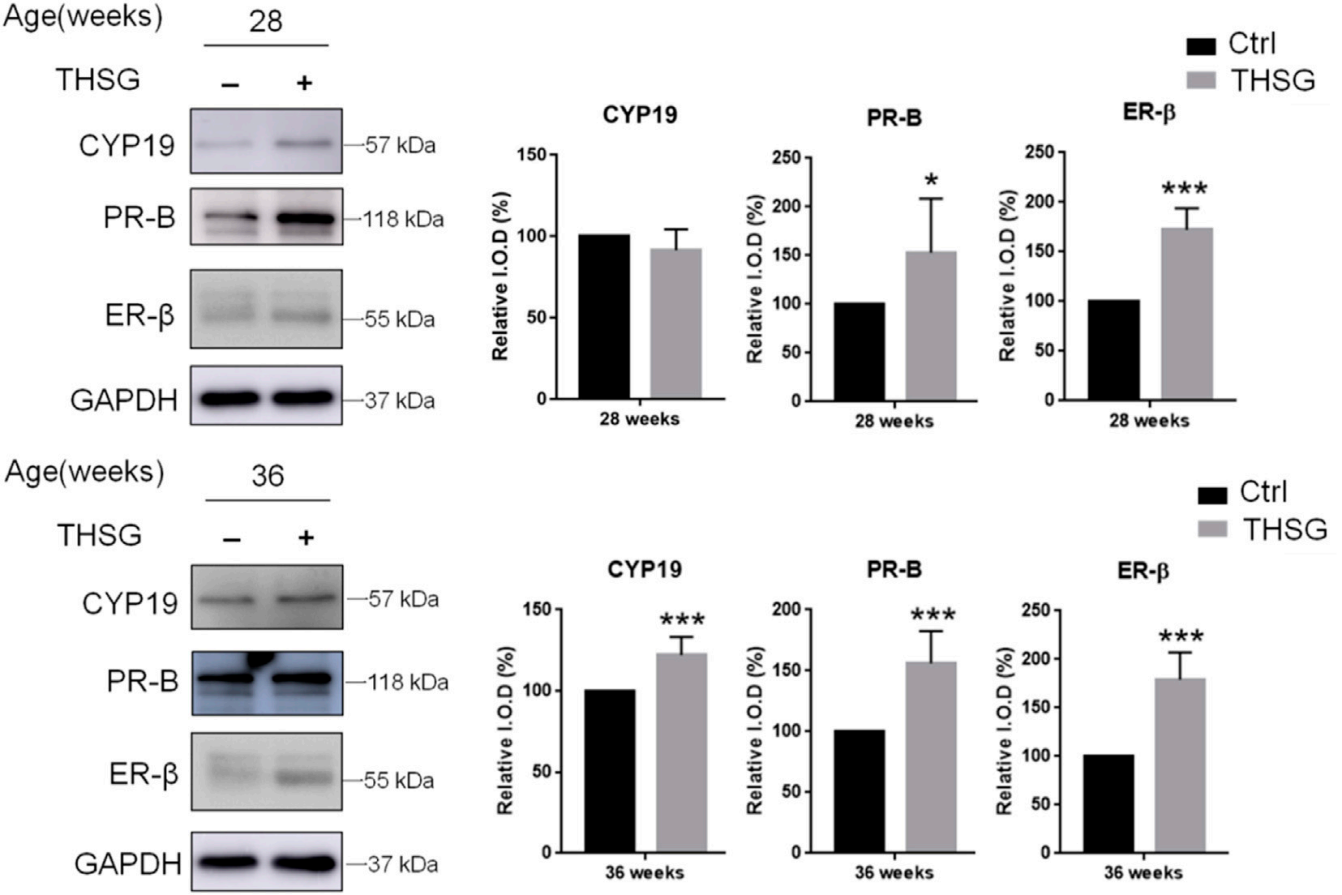
THSG stimulates steroidogenesis-associated proteins in aging young mice. Female C57BL/6J mice (4 weeks age-old) were received 10 mg/kg THSG (in 50% ethanol; experiment group) or solvent (50% ethanol; control group) for 6 weeks (28 weeks age-old) or 8 weeks (36 weeks age-old) *via* gavage feeding. Ovaries of each mouse were collected from oviducts 14 h after hCG administration. Total proteins of oocytes were extracted. Western blotting analyses were conducted for CYP19, PR-B, ER-β and GAPDH. Data are expressed as the mean ± SD; *p < 0.05 and ***p < 0.001, compared with control group.

To determine whether THSG affects oocyte quality, the expression of hormone-related proteins in the ovary was investigated. We examined CYP19, an aromatase enzyme also known as estrogen synthetase or estrogen synthase, and two steroid hormone-related receptors, progesterone receptor-B (PR-B) and estrogen receptor-β (ER-β) (Figure 4). There were differences between control and THSG-treated groups at 28 weeks of age (Start feeding at 4 weeks) and at 36 weeks of age (Start feeding at 4 weeks). The accumulation of PR-B and ER-β was significantly increased in 28-week-old mice after THSG administration. In addition, all of the CYP19, PR-B, and ER-β expressions were significantly increased in 36-week-old mice. The results show that THSG can increase the synthesis and sensitivity of sex hormones, thereby maintaining the quality of oocytes.

### Tetrahydroxystilbene-2-*O*-β-D-Glucoside treatment defers the degradation of oocyte quantity and quality in aged mice groups

**FIGURE 5:**
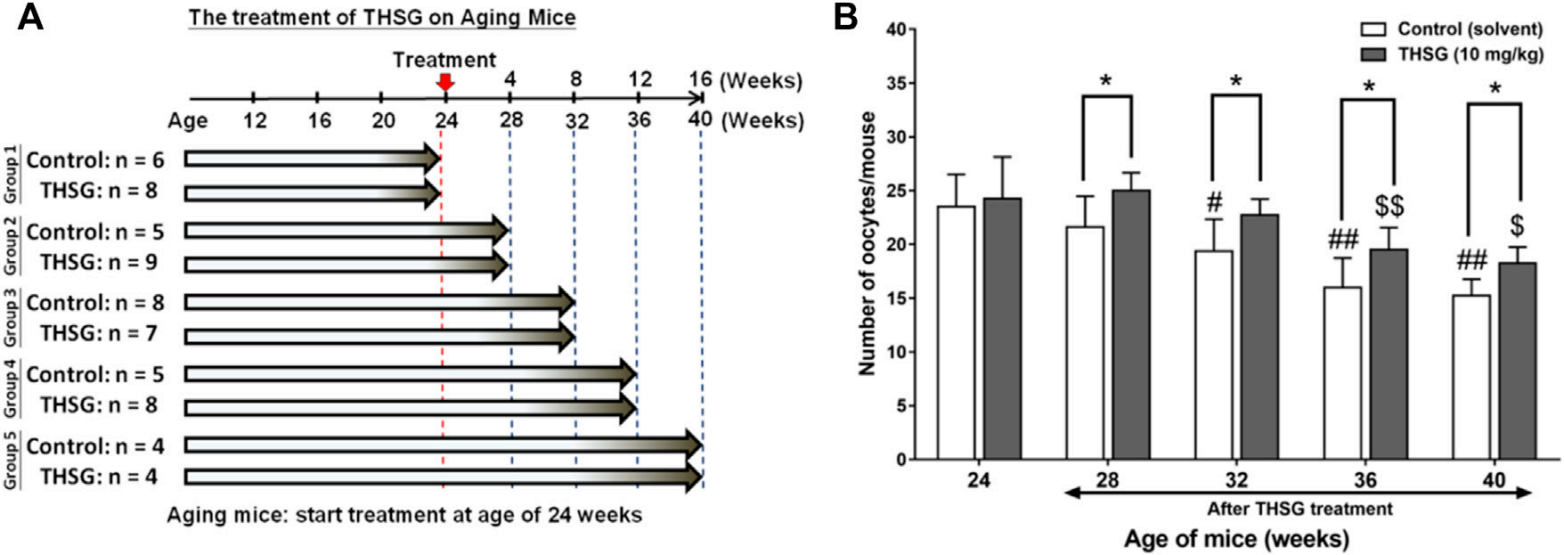
THSG treatment enhances oocyte quantity in aged mice groups. **(A)** Female C57BL/6J mice (24 week-old) were received 10 mg/kg THSG (in 50% ethanol; experiment group) or solvent (50% ethanol; control group) for 16 weeks *via* gavage feeding. **(B)** Oocytes of each mouse were collected from oviducts 14 h after hCG administration. Data are expressed as the mean ± SD; *p < 0.05, compared with each age-matched control group; #*p* < 0.05, ##*p* < 0.01, compared with the control group of 24-week-old mice; $ *p* < 0.05, $$ *p* < 0.01, compared with the THSG-treated group of 24-week-old mice.

The effects of THSG in aged mice were also performed. As shown in Figure 5A, 24-week-old mice were fed THSG 10 mg/kg twice weekly for different periods. Mice were sacrificed at the end of every 4-weeks feeding period, up to a maximum of 40 weeks. The number of oocytes per mouse decreased from 28 weeks of age in the control group but did not decrease until 36 weeks in the THSG-fed group (Figure 5B). There were significant differences between THSG-fed and untreated controls. The results also showed that the percentage of secondary polar bodies (PB2) and activated oocytes decreased at approximately 32 weeks of age in untreated controls (Supplementary Figure S2). However, THSG-fed animals maintained the percentage of PB2 and activated oocytes throughout the study period. THSG also increased the percentage of activated oocytes compared to 24-week-old mice in the aged mice group.

**FIGURE 6:**
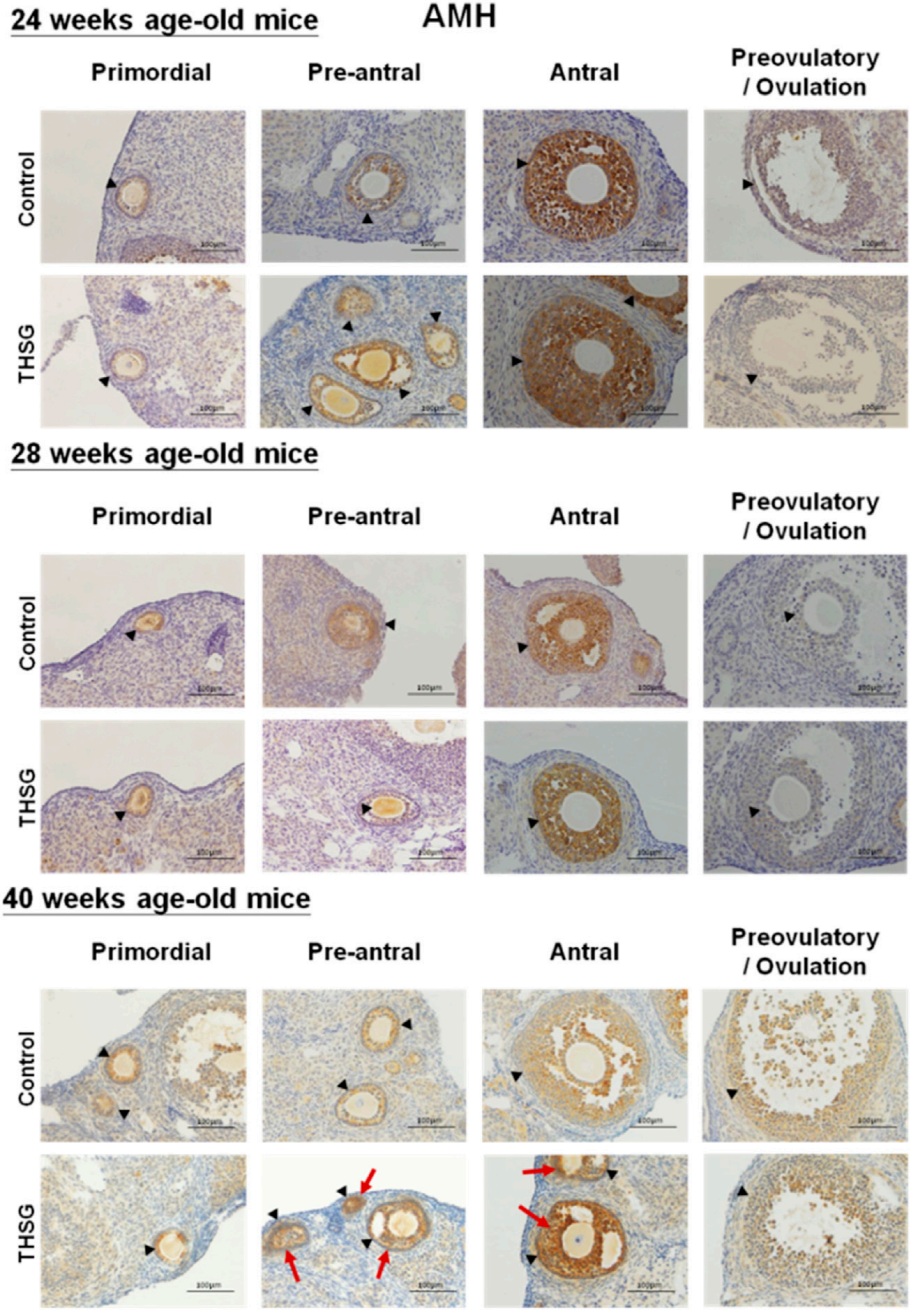
THSG affects protein levels of AMH in different stages of folliculogenesis. The black triangles indicate the follicle formation, and the red arrows indicate the significant AMH accumulation (the brown area). Mice fed with THSG or untreated controls were sacrificed at the end of 24, 28, and 40 weeks of age. IHC of AMH was stained. AMH accumulation showed no difference between the THSG-fed and untreated control groups in 24-week-old and 28-week-old mice. There was more dominant AMH accumulation, especially during the antral follicular phase in THSG-fed elder mice groups (40-week-old mice) than in the control group.

In order to investigate mechanisms involved in THSG-induced protection of oocyte quality in aging and aged mice, immunohistochemical studies (IHC) were conducted to compare morphological characteristics of the ovaries in control and THSG-fed mice. Anti-Müllerian hormone (AMH), also known as Müllerian-inhibiting hormone (MIH), is used as a marker of oocyte quantity and an important factor in regulating follicular development (Rzeszowska et al., 2016; Xu et al., 2016). In addition, AMH has also been used as a biomarker molecule for the relative size of the ovarian reserve (Weenen et al., 2004). The protein accumulation of AMH showed no difference between the THSG-fed and untreated control groups in 24-week-old and 28-week-old mice (Figure 6). However, in the elder mice groups (40-week-old mice), the AMH accumulation in treatment groups was higher than that in the control group, especially during the antral follicular phase (as the red arrow indicated). These results suggest that THSG increased AMH expression in aging mice, which preserved oocyte viability and improved subsequent follicle formation.

### Tetrahydroxystilbene-2-*O*-β-D-Glucoside promotes the gene expression of mitochondrial biogenesis in ovaries of aged mice

**FIGURE 7:**
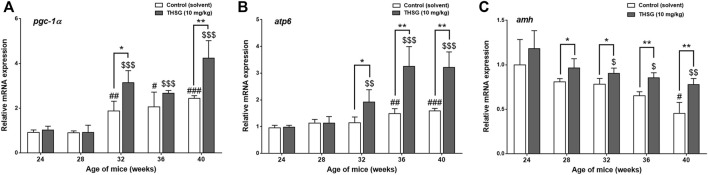
THSG affects gene expression of mitochondrial biogenesis in aged mice groups. Mice fed with THSG or untreated controls were sacrificed at the end of 24, 28, 32, 36, and 40 weeks of age. Ovaries were harvested and total RNA was extracted. qPCR was conducted for *pgc1α* **(A)**, *atp6* **(B)**, and *amh* **(C)**. The number of independent studies (*n*) = 4. Results were expressed as the mean ± SD; *p < 0.05, **p < 0.01, ***p < 0.001, compared with each age-matched control group; #*p* < 0.05, ##*p* < 0.01, ###*p* < 0.001, compared with the control group of 24-week-old mice; $ *p* < 0.05, $$ *p* < 0.01, $$$ *p* < 0.001, compared with the THSG-treated group of 24-week-old mice.

To determine whether the presence of THSG affects mitochondrial activity, the expression of several genes critical for the mitochondrial function was compared in THSG-treated and untreated mice in aged mice (24 weeks of age). PGC1α is a transcriptional coactivator and an important inducer of mitochondrial biogenesis (Popov, 2020). After the 4 weeks of feeding (28 weeks of age), the expression levels of *pgc1α*, were similar between the THSG-fed and control groups (Figure 7A). However, *pgc1α* expression was significantly increased in 32-week-old mice upon THSG treatment compared to 24-week-old mice. THSG significantly increased *pgc1α* expression compared to age-matched control in 32-week-old mice. Expression of this gene was significantly increased in the 32- and 40-week-old study groups, but there was no significant difference between these two groups in either THSG-fed or untreated control (Figure 7A). The *atp6* gene is essential for making one part of ATP synthase. The increase of *atp6* expression was aging-dependent in both control and THSG-fed mice (Figure 7B). It increased significantly after 8 weeks of THSG feeding (32 weeks of age) compared to age-matched control (Figure 7B). There was a significantly increased *atp6* expression in THSG-fed mice than that in age-matched control mice after 12 and 16 weeks of THSG feeding.

The decrease of *amh* expression was age-dependent in aged mice significantly (Figure 7C). In the gene expression results of *amh*, there was no significant difference in *amh* expression between THSG-fed mice and the control group until 8 weeks of feeding (32-week-old mice) (Figure 7C). The *amh* expression in the control group was significantly lower than that in THSG-fed group, although THSG-fed mice had lower *amh* expression in 32-week-old mice than that in 24-week-old mice after 8 weeks of THSG-fed (32-week-old). After 16 weeks of treatment (40-week-old mice), the *amh* expression in the THSG-fed group was 30% higher than in the control group. The gene expression results were similar to the results of IHC in Figure 6.

### Tetrahydroxystilbene-2-*O*-β-D-Glucoside promotes the gene expression of steroidogenic enzymes and steroidogenesis in ovaries of aged mice

**FIGURE 8:**
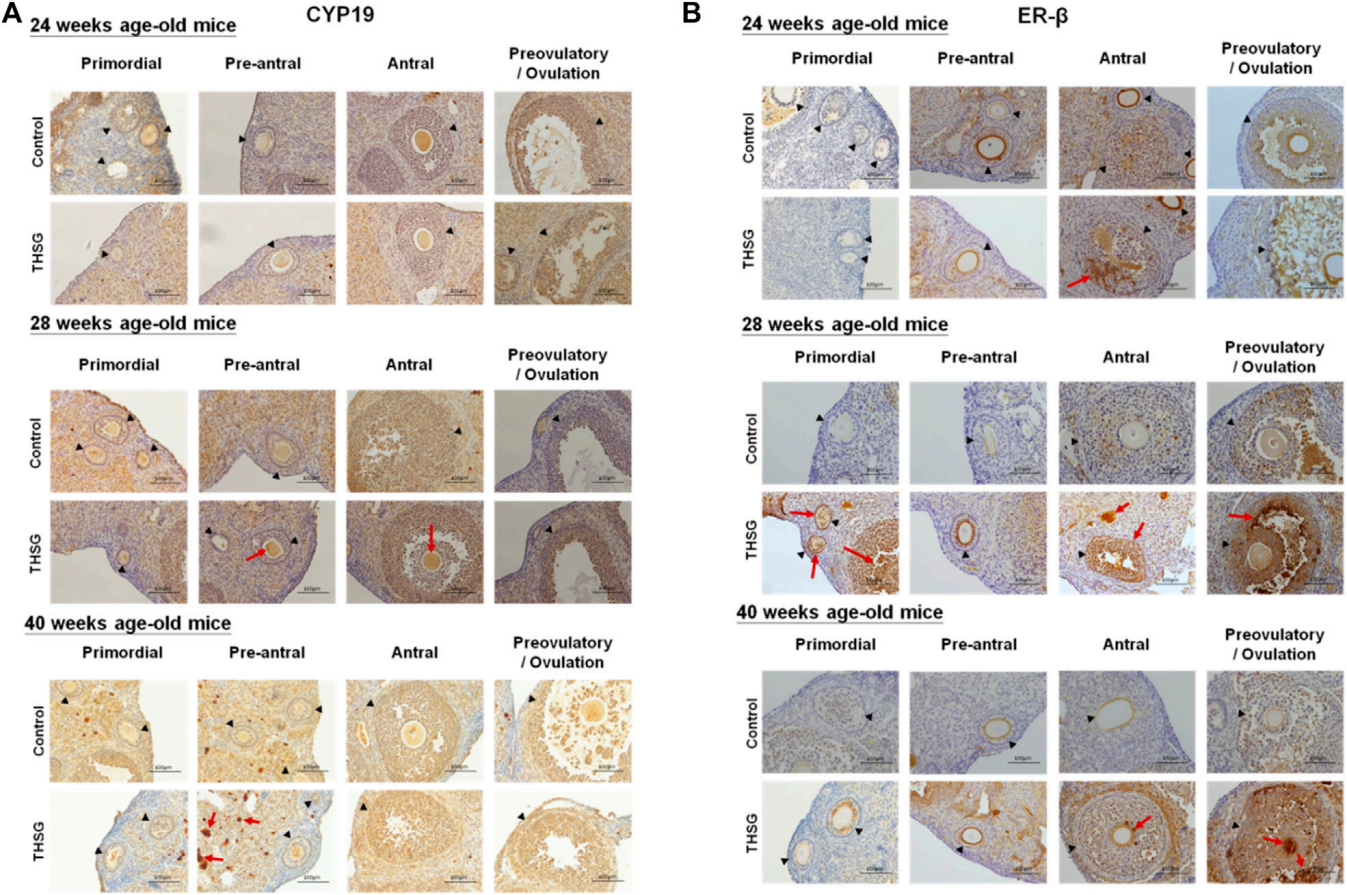
THSG affects protein levels of CYP19 and ER-β in different stages of folliculogenesis. Mice fed with THSG or untreated controls were sacrificed at the end of 24, 28, and 40 weeks of age, and the ovaries were stained by IHC analysis. **(A)** The black triangles indicate the follicle formation, and the red arrows indicate the significant CYP19 accumulation (the brown area). At different stages of follicle maturation, CYP19 accumulation was higher in the THSG-fed group than in the untreated control group. As the mice aged, CYP19 accumulation remained at higher levels in the THSG-treated group compared to the untreated control group. **(B)** The black triangles indicate the follicle formation, and the red arrows indicate the significant ER-β accumulation (the brown area). In THSG-treated mice, ER-β accumulation, especially at the end of 40 weeks, was much higher than in controls.

After 4 weeks of THSG feeding (28 weeks of age), the IHC analysis was conducted to compare CYP19 and ER-β expression in the ovaries of control and THSG-fed mice. The CYP19 accumulation in THSG-fed groups was higher than that in untreated control groups in different stages of follicular maturation (Figure 8A). With aging, the CYP19 accumulation decreased in both groups. However, the fading rate was slower in THSG-treated groups than that in the untreated control. Similar results were observed in ER-β accumulation, especially, at the end of 40 weeks, the ER-β accumulation (as the red arrows indicated) was much higher in THSG-treated mice than that in control groups (Figure 8B). These results suggested that THSG revealed an increase in estrogen-binding capacities by activating the expression of CYP19 and ER-β.

**FIGURE 9:**
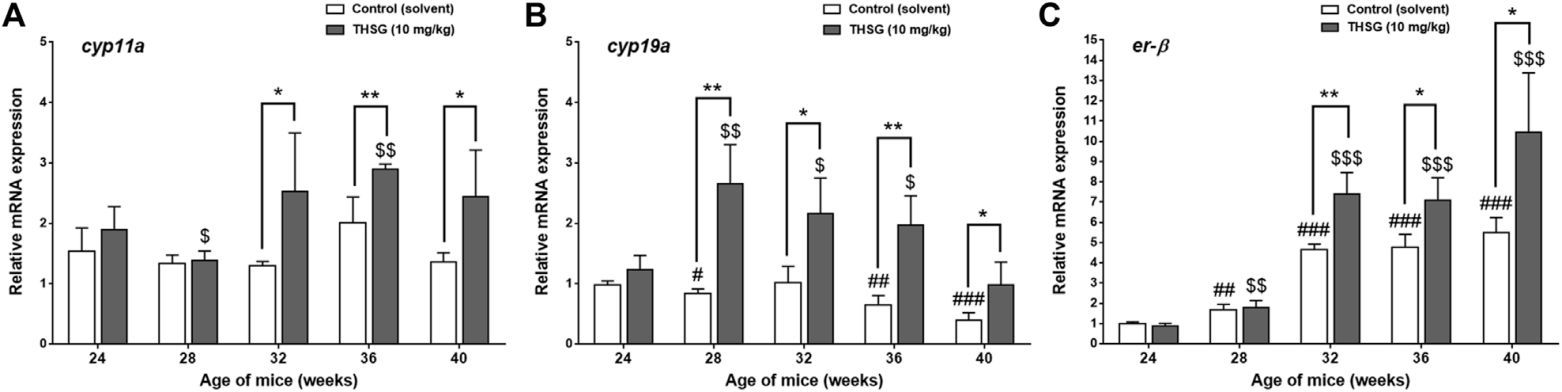
THSG affects gene expression of steroidogenic enzymes and *er-β* in aged mice groups. Mice fed with THSG or untreated controls were sacrificed at the end of 24, 28, 32, 36, and 40 weeks of age. Ovaries were harvested and total RNA was extracted. qPCR was conducted for *cyp11a* **(A)**, *cyp19a* **(B)**, and *er-β* **(C)**. The number of independent studies (*n*) = 4. Results were expressed as the mean ± SD; *p < 0.05, **p < 0.01, compared with each age-matched control group; #*p* < 0.05, ##*p* < 0.01, ###*p* < 0.001, compared with the control group of 24-week-old mice; $ *p* < 0.05, $$ *p* < 0.01, $$$ *p* < 0.001, compared with the THSG-treated group of 24-week-old mice.

There are several gene products involved in the process of steroidogenesis. We examined two of them. Aromatases, *cyp11a*, and *cyp19a*, are also called estrogen synthetases or estrogen synthases (Figures 9A,B). In addition, we investigated the expression of *er-β* gene related to estrogen sensitivity (Figure 9C). There was no difference between control and THSG-treated groups until 32 weeks (after 8 weeks of feeding). The expression of *cyp11a* slightly increased at 36 weeks and dropped again at 40 weeks (Figure 9A). THSG-treatment significantly increased *cyp11a* expression at 32 weeks and maintained a higher level than that in control. Otherwise, THSG stimulated *cyp19a* expression at 28 weeks and maintained a higher level compared to control for the whole experimental period (Figure 9B). The *cyp19a* expression was significantly activated and higher than that in control although the expression decreased gradually. Expression of *er-β* was activated during the aging period and THSG stimulated even more significantly compared to untreated control in the end studies (Figure 9C). Results indicated that THSG may enhance mitochondrial biogenesis and steroidogenesis in aged mice.

## Discussion

Aging increases the problem of infertility. However, the molecular mechanisms by which it affects infertility are complex. Several studies have shown a link between inflammation and infertility (Weiss et al., 2009; Vaisi-Raygani and Asgari, 2021), especially inflammation of the peritoneal cavity (Tao et al., 2018; Mohammed Rasheed and Hamid, 2020). Long-term chronic low-grade inflammation affects female fertility. The ability of natural compounds to modulate the effects of inflammation has recently received considerable attention (Shahidi and Ambigaipalan, 2015). Several antioxidant reagents have been used to prevent or treat infertility, such as resveratrol, coenzyme-Q10, melatonin, folic acid, and several vitamins (Rodríguez-Varela and Labarta, 2020; Tesarik, 2021). Resveratrol exists in many food sources such as grapes, wine, peanuts, soybeans, berries, and stilbenes. Resveratrol inhibits the synthesis of prostaglandins to induce a significant anti-inflammatory effect (Dull et al., 2019). Resveratrol has been shown to exhibit apoptosis-inducing activity (Fu et al., 2021). Resveratrol neutralizes free radicals and oxidizing molecules that contribute to the anti-inflammatory process (Bhat and Pezzuto, 2002). Resveratrol directly inhibits the expression of pro-inflammatory cytokines such as TNF-α, IL-1β, IL-6, IL-10, MCP-1, IFNα, and IFNβ in different tissues in animal models (Knobloch et al., 2011; Yen et al., 2011; Zhu et al., 2011; Mbimba et al., 2012; Zhang et al., 2013a). THSG also has anti-inflammatory effects by inhibiting TNF-α, IL-1β, IL-6, NO production as well as suppressing the expression of cyclooxygenase-2 (COX-2) and inhibiting the NF-κB pathway (Wang et al., 2022). This anti-inflammation may help to retard aging. Resveratrol has the potential therapeutic effect of improving ovarian function (Ochiai and Kuroda, 2020). However, studies indicated that resveratrol also induces anti-deciduogenic actions on the uterine endometrium. In addition, it cannot rule out the possibility of teratogenicity by resveratrol. Thus, resveratrol may be proper to use during the luteal phase and pregnancy. Instead, THSG is glycosylated resveratrol, shown in our previous study to be more effective and safer than resveratrol (Chin et al., 2017).

When mice were fed THSG from 4 weeks of age, THSG did not significantly increase the number of oocytes in 24-week-old mice compared to untreated controls (Figure 2B). Oocytes in elder mice were less than in younger mice. After 28 weeks treated with THSG (32 weeks of age), the number of oocytes was higher than the control group (*p* < 0.05). While the ovaries were confronted with aging, the number of preantral and antral follicles tremendously reduced in the untreated control group (Figure 3C), and there were plenty of preantral (growing) and antral (mature) follicles in the ovaries from THSG-fed mice (Figures 3D–F). The expressions of steroid hormone-associated proteins, PR-B, and ER-β, were significantly higher in THSG-fed 28-week-old mice than in untreated controls (Figure 4). In addition, all expressions of CYP19, PR-B and ER-β were significantly increased in 36-week-old mice fed THSG for 32 weeks. The ovarian aromatase enzyme CYP19 is critical within the steroid biosynthetic pathway for the conversion of androgen precursors into estradiol (Payne and Hales, 2004). THSG-fed 36-week-old mice produced sufficient CYP19, while higher CYP19 expression was detected in THSG-fed mice (Figure 4). The expressions of PR-B and ER-β are related to hormone sensitivity and have been well-defined to impact oocyte quality (Sar and Welsch, 1999; Emmen et al., 2005; Robker et al., 2009; Lonergan, 2011). Thus, THSG augmented the expression of PR-B and ER-β to facilitate steroid receptor sensitivity in groups of young mice that received long-term THSG treatment (fed for at least 24 weeks from 4 weeks of age). THSG was able to slow down the decline of oocyte numbers in aged mice (fed from 24 weeks of age) (Figure 5B). The oocyte numbers per mouse started to drop at 28 weeks in the control group (Figure 5B). However, oocyte numbers did not reduce until 36 weeks with THSG-fed. Differences between control and THSG treatment were significant in mice aged 28–40 weeks. On the other hand, THSG-fed mice had more secondary polar bodies (PB2) and activated oocytes compared to untreated controls (Supplementary Figures S1, S2). These results suggested that THSG can retard aging-related infertility.

Studies by Foote et al. have shown that arterial mitochondrial respiration significantly declines with age, and mitochondrial DNA (mtDNA) integrity and mitochondrial function decline directly accelerate vascular aging (Foote et al., 2018). Although mitochondrial DNA (mtDNA) damage is a predictable factor in aging, it is not clear whether there is mtDNA damage or mitochondrial dysfunction that directly accelerates senescence of the ovaries. Our results indicated that THSG treatment promoted some mitochondrial biosynthetic gene expression, such as *pgc1α* and *atp6* (Figures 7A,B). After THGS treatment, *pgc1α* gene expression was significantly increased in 32- and 40-week-old mice (Figure 7A). Initially, *pgc1α* expression was similar between the THSG-fed and control groups but increased after 8 weeks of THSG treatment (32 weeks of age). THSG significantly increased much more gene expression of *pgc1α* compared to controls in 32-week-old mice. On the other hand, the increase of *atp6* expression in aged mice studies was aging-dependent in both control and THSG-fed mice, however, *atp6* was significantly increased after 8 weeks of THSG treatment (32 weeks of age) (Figure 7B). The *atp6* gene is essential for making one part of ATP synthase, which maintained normal mitochondrial function (Ganetzky et al., 2019). These results suggest that THSG regulated the expression of mitochondrial biogenetic genes to reduce the decay of mitochondrial activity and ROS levels. While *pgc1α* and *atp6* expression increased in the control group with subsequent progression of aging, these aging-prevented effects may be increased significantly by THSG administration. The AMH expression in THSG-fed mice decreased when mice were aging and aged, but remained significantly higher in 32-, 36-, and 40-week-old mice than in controls (Figures 6, 7C). AMH is a substance produced by granulosa cells of female preantral follicles and small antral follicles. It wraps and provides energy to every oocyte. AMH plays a vital role in growth differentiation and follicle formation (Rzeszowska et al., 2016). AMH priming produces more abundant active mitochondria and ATP levels in oocytes (Sinha et al., 2022). Reduced *amh* expression is detected long before normal menopause (Sanders et al., 2009; Seifer et al., 2011) and during the normal aging course in mice with premature ovarian failure (Kevenaar et al., 2006).

Natural menopause is a normal phenomenon of aging (Broekmans et al., 2009). When aging progresses, the reproductive cycle begins to slow down and prepares to stop. Those procedures lead the oocytes to decline in both quantity and quality. Follicle maturation needs some of the gonadotropin and estrogen hormones to stimulate. In addition, gonadotropins need steroidal mediators to function as critical hormones to accomplish oocyte growth and maturation (Senthilkumaran et al., 2004). Moreover, these steroid hormones are synthesized from cholesterol, a precursor involved in the biosynthesis of steroid hormones (Payne and Hales, 2004). Upregulation of cytochrome P450 (CYP) enzyme expression is associated with steroidogenesis. The elevations of steroidogenesis-related gene (*cyp11a* and *cyp19a*) expressions boost the biosynthesis of the gonadal steroid hormones such as progesterone and estradiol, thereby preventing oocyte decline due to aging infertility. In addition to diverse changes in steroidogenesis, steroidogenic enzyme genes appear in the granular layer of ovarian follicles prior to oocyte maturation (Senthilkumaran et al., 2004). On the other hand, the ER-β, a member of the estrogen receptor family, probably plays a vital role in regulating follicle maturation (Sar and Welsch, 1999; Emmen et al., 2005) and gonadotropin secretion (Lee et al., 2021). The immunohistochemistry (IHC) analysis was conducted to confirm the protein levels of CYP19 and ER-β expression in the ovaries of control and THSG-fed mice (Figure 8). After THSG treatment, both CYP19 and ER-β were higher in different stages of follicle maturation than in the untreated group. THSG treatment increased the expression of genes associated with steroidogenesis (*cyp11a* and *cyp19a*) (Figures 9A,B). The expression of *cyp11a* increased significantly at 32 weeks (8 weeks after feeding) and remained above the control level. THSG stimulated *cyp19a* expression at 28 weeks (4 weeks after feeding), although *cyp19a* expression gradually decreased due to aging. The age-related changes in circulating estrogen have broad consequences on ovarian. The amount of ER-β also decreases with aging, which might be correlated with a decline in estrogen sensitivity. Previous studies have indicated that ER-β is essential for normal ovarian function (Sar and Welsch, 1999; Emmen et al., 2005). Our findings also show that THSG promotes and maintains *er-β* expression at high levels (Figure 9C). These gene expression results were partially consistent with the corresponding IHC analysis in CYP19 and ER-β. These outcomes suggest that THSG may promote estrogen synthesis and enhance the binding capacities of estrogen receptors. Herein, the THSG of *Polygonum multiflorum* extract could stimulate the maturation of follicles and oocytes and defer the degradation of oocyte quantity and quality. In summary, THSG protected intact oocytes and reduced the decay rate of oocytes in aging mice. Additionally, THSG promoted the expression of genes involved in steroidogenesis, and mitochondrial respiration, thereby increasing the cellular viability of oocytes. These results suggest that THSG administration has a potential therapeutic effect for improving ovarian aging.

## Conclusion

In this study, THSG increased the number of active oocytes in groups of young and aged mice compared to untreated controls. Furthermore, THSG elevated the expression of *atp6* and *pgc1α* genes associated with mitochondrial biogenesis as well as the *cyp11a* and *cyp19a* genes associated with steroidogenesis in ovaries. Consequentially, THSG treatment not only reduced the attenuation of mitochondrial activity but also stimulated steroidogenesis of sex hormones in aging ovaries. THSG was able to slow down the rate of decline in oocyte number in aging and aged mice. These results indicated that THSG protected the ovary from aging stress damage by enhancing the expression of mitochondrial genes related to metabolism and energy. In addition, THSG can also promote the expression of genes related to the production and sensitivity of sex hormones. In conclusion, our findings suggest that THSG may thus be a potential therapeutic agent for the prevention of aging-induced infertility.

## Data Availability

The original contributions presented in the study are included in the article/Supplementary Material, further inquiries can be directed to the corresponding author.

## References

[B1] Agarwal A. Aponte-Mellado A. Premkumar B. J. Shaman A. Gupta S. (2012). The effects of oxidative stress on female reproduction: A review. Reprod. Biol. Endocrinol. 10, 49. 10.1186/1477-7827-10-49 22748101 PMC3527168

[B2] Agarwal A. Gupta S. Sikka S. (2006). The role of free radicals and antioxidants in reproduction. Curr. Opin. Obstet. Gynecol. 18, 325–332. 10.1097/01.gco.0000193003.58158.4e 16735834

[B3] Bedaiwy M. A. Falcone T. Mohamed M. S. Aleem A. A. Sharma R. K. Worley S. E. (2004). Differential growth of human embryos *in vitro*: Role of reactive oxygen species. Fertil. Steril. 82, 593–600. 10.1016/j.fertnstert.2004.02.121 15374701

[B4] Bhat K. P. Pezzuto J. M. (2002). Cancer chemopreventive activity of resveratrol. Ann. N. Y. Acad. Sci. 957, 210–229. 10.1111/j.1749-6632.2002.tb02918.x 12074974

[B5] Bhatt S. R. Lokhandwala M. F. Banday A. A. (2011). Resveratrol prevents endothelial nitric oxide synthase uncoupling and attenuates development of hypertension in spontaneously hypertensive rats. Eur. J. Pharmacol. 667, 258–264. 10.1016/j.ejphar.2011.05.026 21640096

[B6] Brito P. Almeida L. M. Dinis T. C. (2002). The interaction of resveratrol with ferrylmyoglobin and peroxynitrite; protection against LDL oxidation. Free Radic. Res. 36, 621–631. 10.1080/10715760290029083 12180187

[B7] Broekmans F. J. Soules M. R. Fauser B. C. (2009). Ovarian aging: Mechanisms and clinical consequences. Endocr. Rev. 30, 465–493. 10.1210/er.2009-0006 19589949

[B8] Chaube S. K. Prasad P. V. Thakur S. C. Shrivastav T. G. (2005). Hydrogen peroxide modulates meiotic cell cycle and induces morphological features characteristic of apoptosis in rat oocytes cultured *in vitro* . Apoptosis 10, 863–874. 10.1007/s10495-005-0367-8 16133876

[B9] Chen Y.-F. Yang Y.-N. Chu H.-R. Huang T.-Y. Wang S.-H. Chen H.-Y. (2022). Role of integrin αvβ3 in doxycycline-induced anti-proliferation in breast cancer cells. Front. Cell Dev. Biol. 10, 829788. 10.3389/fcell.2022.829788 35237605 PMC8884148

[B10] Chin Y.-T. Cheng G.-Y. Shih Y.-J. Lin C.-Y. Lin S.-J. Lai H.-Y. (2017). Therapeutic applications of resveratrol and its derivatives on periodontitis. Ann. N. Y. Acad. Sci. 1403, 101–108. 10.1111/nyas.13433 28856691

[B11] Chin Y.-T. Hsieh M.-T. Lin C.-Y. Kuo P.-J. Yang Y.-C. S. H. Shih Y.-J. (2016). 2, 3, 5, 4′-tetrahydroxystilbene-2-O-β-glucoside isolated from Polygoni multiflori ameliorates the development of periodontitis. Mediat. Inflamm. 2016, 6953459. 10.1155/2016/6953459 PMC4967694 27504055

[B12] Chin Y.-T. Wei P.-L. Ho Y. Nana A. W. Changou C. A. Chen Y.-R. (2018). Thyroxine inhibits resveratrol-caused apoptosis by PD-L1 in ovarian cancer cells. Endocr. Relat. Cancer 25, 533–545. 10.1530/ERC-17-0376 29555649

[B13] Choi W. J. Banerjee J. Falcone T. Bena J. Agarwal A. Sharma R. K. (2007). Oxidative stress and tumor necrosis factor-alpha-induced alterations in metaphase II mouse oocyte spindle structure. Fertil. Steril. 88, 1220–1231. 10.1016/j.fertnstert.2007.02.067 17601599

[B14] Das S. Chattopadhyay R. Ghosh S. Ghosh S. Goswami S. K. Chakravarty B. N. (2006). Reactive oxygen species level in follicular fluid--embryo quality marker in IVF? Hum. Reprod. 21, 2403–2407. 10.1093/humrep/del156 16861701

[B15] De Koning C. H. Popp-Snijders C. Schoemaker J. Lambalk C. B. (2000). Elevated FSH concentrations in imminent ovarian failure are associated with higher FSH and LH pulse amplitude and response to GnRH. Hum. Reprod. 15, 1452–1456. 10.1093/humrep/15.7.1452 10875849

[B16] Den Tonkelaar I. Te Velde E. R. Looman C. W. (1998). Menstrual cycle length preceding menopause in relation to age at menopause. Maturitas 29, 115–123. 10.1016/s0378-5122(98)00013-9 9651900

[B17] Dull A. M. Moga M. A. Dimienescu O. G. Sechel G. Burtea V. Anastasiu C. V. (2019). Therapeutic approaches of resveratrol on endometriosis via anti-inflammatory and anti-angiogenic pathways. Molecules 24, E667. 10.3390/molecules24040667 30781885 PMC6413140

[B18] Eghbaliferiz S. Iranshahi M. (2016). Prooxidant activity of polyphenols, flavonoids, anthocyanins and carotenoids: Updated review of mechanisms and catalyzing metals. Phytother. Res. 30, 1379–1391. 10.1002/ptr.5643 27241122

[B19] Eini F. Bidadkosh A. Nazarian H. Piryaei A. Ghaffari Novin M. Joharchi K. (2019). Thymoquinone reduces intracytoplasmic oxidative stress and improves epigenetic modification in polycystic ovary syndrome mice oocytes, during *in-vitro* maturation. Mol. Reprod. Dev. 86, 1053–1066. 10.1002/mrd.23222 31209968

[B20] Elizur S. E. Lebovitz O. Orvieto R. Dor J. Zan-Bar T. (2014). Reactive oxygen species in follicular fluid may serve as biochemical markers to determine ovarian aging and follicular metabolic age. Gynecol. Endocrinol. 30, 705–707. 10.3109/09513590.2014.924100 25014488

[B21] Emmen J. M. Couse J. F. Elmore S. A. Yates M. M. Kissling G. E. Korach K. S. (2005). *In vitro* growth and ovulation of follicles from ovaries of estrogen receptor (ER){alpha} and ER{beta} null mice indicate a role for ER{beta} in follicular maturation. Endocrinology 146, 2817–2826. 10.1210/en.2004-1108 15731357

[B22] Foote K. Reinhold J. Yu E. P. K. Figg N. L. Finigan A. Murphy M. P. (2018). Restoring mitochondrial DNA copy number preserves mitochondrial function and delays vascular aging in mice. Aging Cell 17, e12773. 10.1111/acel.12773 29745022 PMC6052475

[B23] Fritz R. Jindal S. (2018). Reproductive aging and elective fertility preservation. J. Ovarian Res. 11, 66. 10.1186/s13048-018-0438-4 30098598 PMC6087539

[B24] Fu X. Li M. Tang C. Huang Z. Najafi M. (2021). Targeting of cancer cell death mechanisms by resveratrol: A review. Apoptosis. 26, 561–573. 10.1007/s10495-021-01689-7 34561763

[B25] Fujii J. Iuchi Y. Okada F. (2005). Fundamental roles of reactive oxygen species and protective mechanisms in the female reproductive system. Reprod. Biol. Endocrinol. 3, 43. 10.1186/1477-7827-3-43 16137335 PMC1224869

[B26] Ganetzky R. D. Stendel C. Mccormick E. M. Zolkipli-Cunningham Z. Goldstein A. C. Klopstock T. (2019). MT-ATP6 mitochondrial disease variants: Phenotypic and biochemical features analysis in 218 published cases and cohort of 14 new cases. Hum. Mutat. 40, 499–515. 10.1002/humu.23723 30763462 PMC6506718

[B27] Gleicher N. Weghofer A. Barad D. H. (2010). Discordances between follicle stimulating hormone (FSH) and anti-Müllerian hormone (AMH) in female infertility. Reprod. Biol. Endocrinol. 8, 64. 10.1186/1477-7827-8-64 20565808 PMC2894827

[B28] Gregory S. J. Kaiser U. B. (2004). Regulation of gonadotropins by inhibin and activin. Semin. Reprod. Med. 22, 253–267. 10.1055/s-2004-831901 15319828

[B29] Grive K. J. Freiman R. N. (2015). The developmental origins of the mammalian ovarian reserve. Development 142, 2554–2563. 10.1242/dev.125211 26243868 PMC4529035

[B30] Gurer-Orhan H. Suzen S. (2015). Melatonin, its metabolites and its synthetic analogs as multi-faceted compounds: Antioxidant, prooxidant and inhibitor of bioactivation reactions. Curr. Med. Chem. 22, 490–499. 10.2174/0929867321666141215095259 25515518

[B31] Ho Y. Wu C.-Y. Chin Y.-T. Li Z.-L. Pan Y.-S. Huang T.-Y. (2020). NDAT suppresses pro-inflammatory gene expression to enhance resveratrol-induced anti-proliferation in oral cancer cells. Food Chem. Toxicol. 136, 111092. 10.1016/j.fct.2019.111092 31883986

[B32] Huang C. Wang Y. Wang J. Yao W. Chen X. Zhang W. (2013). TSG (2, 3, 4’ , 5-tetrahydroxystilbene 2-O-β-D-glucoside) suppresses induction of pro-inflammatory factors by attenuating the binding activity of nuclear factor-κB in microglia. J. Neuroinflammation 10, 129. 10.1186/1742-2094-10-129 24144353 PMC3854509

[B33] Jiang P. Du W. Mancuso A. Wellen K. E. Yang X. (2013). Reciprocal regulation of p53 and malic enzymes modulates metabolism and senescence. Nature 493, 689–693. 10.1038/nature11776 23334421 PMC3561500

[B34] Kevenaar M. E. Meerasahib M. F. Kramer P. Van De Lang-Born B. M. De Jong F. H. Groome N. P. (2006). Serum anti-mullerian hormone levels reflect the size of the primordial follicle pool in mice. Endocrinology 147, 3228–3234. 10.1210/en.2005-1588 16556768

[B35] Klein N. A. Battaglia D. E. Fujimoto V. Y. Davis G. S. Bremner W. J. Soules M. R. (1996). Reproductive aging: Accelerated ovarian follicular development associated with a monotropic follicle-stimulating hormone rise in normal older women. J. Clin. Endocrinol. Metab. 81, 1038–1045. 10.1210/jcem.81.3.8772573 8772573

[B36] Klein N. A. Harper A. J. Houmard B. S. Sluss P. M. Soules M. R. (2002). Is the short follicular phase in older women secondary to advanced or accelerated dominant follicle development? J. Clin. Endocrinol. Metab. 87, 5746–5750. 10.1210/jc.2002-020622 12466381

[B37] Knobloch J. Hag H. Jungck D. Urban K. Koch A. (2011). Resveratrol impairs the release of steroid-resistant cytokines from bacterial endotoxin-exposed alveolar macrophages in chronic obstructive pulmonary disease. Basic Clin. Pharmacol. Toxicol. 109, 138–143. 10.1111/j.1742-7843.2011.00707.x 21447053

[B38] Lee E. B. Chakravarthi V. P. Wolfe M. W. Karim Rumi M. A. (2021). ERβ regulation of gonadotropin responses during folliculogenesis. Int. J. Mol. Sci. 22, 10348. 10.3390/ijms221910348 34638689 PMC8508937

[B39] Lee S. Jeong S. Y. Lim W. C. Kim S. Park Y. Y. Sun X. (2007). Mitochondrial fission and fusion mediators, hFis1 and OPA1, modulate cellular senescence. J. Biol. Chem. 282, 22977–22983. 10.1074/jbc.M700679200 17545159

[B40] Lim J. Luderer U. (2011). Oxidative damage increases and antioxidant gene expression decreases with aging in the mouse ovary. Biol. Reprod. 84, 775–782. 10.1095/biolreprod.110.088583 21148108 PMC3062040

[B41] Ling S. Xu J.-W. (2016). Biological activities of 2, 3, 5, 4′-tetrahydroxystilbene-2-O-β-D-Glucoside in antiaging and antiaging-related disease treatments. Oxid. Med. Cell. Longev. 2016, 4973239. 10.1155/2016/4973239 27413420 PMC4931083

[B42] Liu L. Keefe D. L. (2002). Ageing-associated aberration in meiosis of oocytes from senescence-accelerated mice. Hum. Reprod. 17, 2678–2685. 10.1093/humrep/17.10.2678 12351548

[B43] Liu M.-J. Sun A.-G. Zhao S.-G. Liu H. Ma S.-Y. Li M. (2018). Resveratrol improves *in vitro* maturation of oocytes in aged mice and humans. Fertil. Steril. 109, 900–907. 10.1016/j.fertnstert.2018.01.020 29778389

[B44] Liu M. Yin Y. Ye X. Zeng M. Zhao Q. Keefe D. L. (2013). Resveratrol protects against age-associated infertility in mice. Hum. Reprod. 28, 707–717. 10.1093/humrep/des437 23293221

[B45] Lonergan P. (2011). Influence of progesterone on oocyte quality and embryo development in cows. Theriogenology 76, 1594–1601. 10.1016/j.theriogenology.2011.06.012 21855985

[B46] Ma S. Feng J. Zhang R. Chen J. Han D. Li X. (2017). SIRT1 activation by resveratrol alleviates cardiac dysfunction via mitochondrial regulation in diabetic cardiomyopathy mice. Oxid. Med. Cell. Longev. 2017, 4602715. 10.1155/2017/4602715 28883902 PMC5572590

[B47] Mbimba T. Awale P. Bhatia D. Geldenhuys W. J. Darvesh A. S. Carroll R. T. (2012). Alteration of hepatic proinflammatory cytokines is involved in the resveratrol-mediated chemoprevention of chemically-induced hepatocarcinogenesis. Curr. Pharm. Biotechnol. 13, 229–234. 10.2174/138920112798868575 21466437

[B48] Mctavish K. J. Jimenez M. Walters K. A. Spaliviero J. Groome N. P. Themmen A. P. (2007). Rising follicle-stimulating hormone levels with age accelerate female reproductive failure. Endocrinology 148, 4432–4439. 10.1210/en.2007-0046 17540727

[B49] Mihalas B. P. Redgrove K. A. Mclaughlin E. A. Nixon B. (2017). Molecular mechanisms responsible for increased vulnerability of the ageing oocyte to oxidative damage. Oxid. Med. Cell. Longev. 2017, 4015874. 10.1155/2017/4015874 29312475 PMC5664291

[B50] Miyamoto K. Sato E. F. Kasahara E. Jikumaru M. Hiramoto K. Tabata H. (2010). Effect of oxidative stress during repeated ovulation on the structure and functions of the ovary, oocytes, and their mitochondria. Free Radic. Biol. Med. 49, 674–681. 10.1016/j.freeradbiomed.2010.05.025 20621580

[B51] Mohammed Rasheed H. A. Hamid P. (2020). Inflammation to infertility: Panoramic view on endometriosis. Cureus 12, e11516. 10.7759/cureus.11516 33354460 PMC7746006

[B52] Moiseeva O. Bourdeau V. Roux A. Deschênes-Simard X. Ferbeyre G. (2009). Mitochondrial dysfunction contributes to oncogene-induced senescence. Mol. Cell. Biol. 29, 4495–4507. 10.1128/MCB.01868-08 19528227 PMC2725737

[B53] Myers M. Britt K. L. Wreford N. G. Ebling F. J. Kerr J. B. (2004). Methods for quantifying follicular numbers within the mouse ovary. Reproduction 127, 569–580. 10.1530/rep.1.00095 15129012

[B54] Nestler J. E. (1998). Inositolphosphoglycans (IPGs) as mediators of insulin's steroidogenic actions. J. Basic Clin. Physiol. Pharmacol. 9, 197–204. 10.1515/jbcpp.1998.9.2-4.197 10212834

[B55] Nuñez-Calonge R. Cortés S. Gutierrez Gonzalez L. M. Kireev R. Vara E. Ortega L. (2016). Oxidative stress in follicular fluid of young women with low response compared with fertile oocyte donors. Reprod. Biomed. Online 32, 446–456. 10.1016/j.rbmo.2015.12.010 26805046

[B56] Ochiai A. Kuroda K. (2020). Preconception resveratrol intake against infertility: Friend or foe? Reprod. Med. Biol. 19, 107–113. 10.1002/rmb2.12303 32273814 PMC7138940

[B57] Payne A. H. Hales D. B. (2004). Overview of steroidogenic enzymes in the pathway from cholesterol to active steroid hormones. Endocr. Rev. 25, 947–970. 10.1210/er.2003-0030 15583024

[B58] Pecyna P. Wargula J. Murias M. Kucinska M. (2020). More than resveratrol: New insights into stilbene-based compounds. Biomolecules 10, 1111. 10.3390/biom10081111 PMC7465418 32726968

[B59] Popov L. D. (2020). Mitochondrial biogenesis: An update. J. Cell. Mol. Med. 24, 4892–4899. 10.1111/jcmm.15194 32279443 PMC7205802

[B60] Richardson M. C. Guo M. Fauser B. C. Macklon N. S. (2014). Environmental and developmental origins of ovarian reserve. Hum. Reprod. Update 20, 353–369. 10.1093/humupd/dmt057 24287894

[B61] Robker R. L. Akison L. K. Russell D. L. (2009). Control of oocyte release by progesterone receptor-regulated gene expression. Nucl. Recept. Signal. 7, e012. 10.1621/nrs.07012 20087433 PMC2807638

[B62] Rodríguez-Varela C. Labarta E. (2020). Clinical application of antioxidants to improve human oocyte mitochondrial function: A review. Antioxidants (Basel) 9, 1197. 10.3390/antiox9121197 PMC7761442 33260761

[B63] Roy H. Nath B. (2021). Antioxidants in female reproductive Biology, antioxidants - benefits, sources, mechanisms of action. London, United Kingdom: IntechOpen Limited. 10.5772/intechopen.95937

[B64] Rzeszowska M. Leszcz A. Putowski L. Hałabiś M. Tkaczuk-Włach J. Kotarski J. (2016). Anti-müllerian hormone: Structure, properties and appliance. Ginekol. Pol. 87, 669–674. 10.5603/GP.2016.0064 27723076

[B65] Salminen A. Kaarniranta K. Hiltunen M. Kauppinen A. (2014). Krebs cycle dysfunction shapes epigenetic landscape of chromatin: Novel insights into mitochondrial regulation of aging process. Cell. Signal. 26, 1598–1603. 10.1016/j.cellsig.2014.03.030 24704120

[B66] Sanders R. D. Spencer J. B. Epstein M. P. Pollak S. V. Vardhana P. A. Lustbader J. W. (2009). Biomarkers of ovarian function in girls and women with classic galactosemia. Fertil. Steril. 92, 344–351. 10.1016/j.fertnstert.2008.04.060 18684449 PMC2746959

[B67] Sar M. Welsch F. (1999). Differential expression of estrogen receptor-beta and estrogen receptor-alpha in the rat ovary. Endocrinology 140, 963–971. 10.1210/endo.140.2.6533 9927330

[B68] Sasaki H. Hamatani T. Kamijo S. Iwai M. Kobanawa M. Ogawa S. (2019). Impact of oxidative stress on age-associated decline in oocyte developmental competence. Front. Endocrinol. 10, 811. 10.3389/fendo.2019.00811 PMC6882737 31824426

[B69] Seifer D. B. Baker V. L. Leader B. (2011). Age-specific serum anti-Mullerian hormone values for 17, 120 women presenting to fertility centers within the United States. Fertil. Steril. 95, 747–750. 10.1016/j.fertnstert.2010.10.011 21074758

[B70] Senthilkumaran B. Yoshikuni M. Nagahama Y. (2004). A shift in steroidogenesis occurring in ovarian follicles prior to oocyte maturation. Mol. Cell. Endocrinol. 215, 11–18. 10.1016/j.mce.2003.11.012 15026170

[B71] Seo A. Y. Joseph A. M. Dutta D. Hwang J. C. Aris J. P. Leeuwenburgh C. (2010). New insights into the role of mitochondria in aging: Mitochondrial dynamics and more. J. Cell Sci. 123, 2533–2542. 10.1242/jcs.070490 20940129 PMC2912461

[B72] Shahidi F. Ambigaipalan P. (2015). Phenolics and polyphenolics in foods, beverages and spices: Antioxidant activity and health effects – a review. J. Funct. Foods 18, 820–897. 10.1016/j.jff.2015.06.018

[B73] Sinha N. Driscoll C. S. Qi W. Huang B. Roy S. Knott J. G. (2022). Anti-Müllerian hormone treatment enhances oocyte quality, embryonic development and live birth rate. Biol. Reprod., ioac116. 10.1093/biolre/ioac116 35657015 PMC9476226

[B74] Soufi F. G. Sheervalilou R. Vardiani M. Khalili M. Alipour M. R. (2012). Chronic resveratrol administration has beneficial effects in experimental model of type 2 diabetic rats. Endocr. Regul. 46, 83–90. 10.4149/endo_2012_02_83 22540856

[B75] Stöckl P. Zankl C. Hütter E. Unterluggauer H. Laun P. Heeren G. (2007). Partial uncoupling of oxidative phosphorylation induces premature senescence in human fibroblasts and yeast mother cells. Free Radic. Biol. Med. 43, 947–958. 10.1016/j.freeradbiomed.2007.06.005 17697939

[B76] Sun Y.-L. Tang S.-B. Shen W. Yin S. Sun Q.-Y. (2019). Roles of resveratrol in improving the quality of postovulatory aging oocytes *in vitro* . Cells 8, 1132. 10.3390/cells8101132 PMC6829324 31547622

[B77] Takeo S. Kimura K. Shirasuna K. Kuwayama T. Iwata H. (2017). Age-associated deterioration in follicular fluid induces a decline in bovine oocyte quality. Reprod. Fertil. Dev. 29, 759–767. 10.1071/RD15228 26829061

[B78] Tamura H. Takasaki A. Miwa I. Taniguchi K. Maekawa R. Asada H. (2008). Oxidative stress impairs oocyte quality and melatonin protects oocytes from free radical damage and improves fertilization rate. J. Pineal Res. 44, 280–287. 10.1111/j.1600-079X.2007.00524.x 18339123

[B79] Tao X. Ge S.-Q. Chen L. Cai L.-S. Hwang M.-F. Wang C.-L. (2018). Relationships between female infertility and female genital infections and pelvic inflammatory disease: A population-based nested controlled study. Clin. Sao Paulo, Braz. 73, e364. 10.6061/clinics/2018/e364 PMC6077933 30110069

[B80] Tesarik J. (2021). Towards personalized antioxidant use in female infertility: Need for more molecular and clinical studies. Biomedicines 9, 1933. 10.3390/biomedicines9121933 34944748 PMC8698668

[B81] Vaisi-Raygani A. Asgari R. (2021). Association of inflammation with female reproductive system disorders. Central Asian J. Med. Pharm. Sci. Innovation 1, 67–73.

[B82] Valeri C. Lovaisa M. M. Racine C. Edelsztein N. Y. Riggio M. Giulianelli S. (2020). Molecular mechanisms underlying AMH elevation in hyperoestrogenic states in males. Sci. Rep. 10, 15062. 10.1038/s41598-020-71675-7 32934281 PMC7492256

[B83] Walczak-Jedrzejowska R. Wolski J. K. Slowikowska-Hilczer J. (2013). The role of oxidative stress and antioxidants in male fertility. Cent. Eur. J. Urol. 66, 60–67. 10.5173/ceju.2013.01.art19 PMC3921845 24578993

[B84] Wang C. Dai S. Gong L. Fu K. Ma C. Liu Y. (2022). A review of pharmacology, toxicity and pharmacokinetics of 2, 3, 5, 4′-tetrahydroxystilbene-2-O-β-D-Glucoside. Front. Pharmacol. 12, 1–23. 10.3389/fphar.2021.791214 PMC8769241 35069206

[B85] Wang F. Tian X. Zhang L. He C. Ji P. Li Y. (2014). Beneficial effect of resveratrol on bovine oocyte maturation and subsequent embryonic development after *in vitro* fertilization. Fertil. Steril. 101, 577–586. e571. 10.1016/j.fertnstert.2013.10.041 24314921

[B86] Wang T. Gu J. Wu P. F. Wang F. Xiong Z. Yang Y. J. (2009). Protection by tetrahydroxystilbene glucoside against cerebral ischemia: Involvement of JNK, SIRT1, and NF-kappaB pathways and inhibition of intracellular ROS/RNS generation. Free Radic. Biol. Med. 47, 229–240. 10.1016/j.freeradbiomed.2009.02.027 19272442

[B87] Wang W. Yang X. López De Silanes I. Carling D. Gorospe M. (2003). Increased AMP:ATP ratio and AMP-activated protein kinase activity during cellular senescence linked to reduced HuR function. J. Biol. Chem. 278, 27016–27023. 10.1074/jbc.M300318200 12730239

[B88] Wang X. De Rivero Vaccari J. P. Wang H. Diaz P. German R. Marcillo A. E. (2012). Activation of the nuclear factor E2-related factor 2/antioxidant response element pathway is neuroprotective after spinal cord injury. J. Neurotrauma 29, 936–945. 10.1089/neu.2011.1922 21806470 PMC3303102

[B89] Wang X. Zhao L. Han T. Chen S. Wang J. (2008). Protective effects of 2, 3, 5, 4'-tetrahydroxystilbene-2-O-beta-d-glucoside, an active component of Polygonum multiflorum Thunb, on experimental colitis in mice. Eur. J. Pharmacol. 578, 339–348. 10.1016/j.ejphar.2007.09.013 17963744

[B90] Wathes D. C. Abayasekara D. R. Aitken R. J. (2007). Polyunsaturated fatty acids in male and female reproduction. Biol. Reprod. 77, 190–201. 10.1095/biolreprod.107.060558 17442851

[B91] Weenen C. Laven J. S. E. Von Bergh A. R. M. Cranfield M. Groome N. P. Visser J. A. (2004). Anti‐müllerian hormone expression pattern in the human ovary: Potential implications for initial and cyclic follicle recruitment. Mol. Hum. Reprod. 10, 77–83. 10.1093/molehr/gah015 14742691

[B92] Weiss G. Goldsmith L. T. Taylor R. N. Bellet D. Taylor H. S. (2009). Inflammation in reproductive disorders. Reprod. Sci. 16, 216–229. 10.1177/1933719108330087 19208790 PMC3107847

[B93] Wiel C. Lallet-Daher H. Gitenay D. Gras B. Le Calvé B. Augert A. (2014). Endoplasmic reticulum calcium release through ITPR2 channels leads to mitochondrial calcium accumulation and senescence. Nat. Commun. 5, 3792. 10.1038/ncomms4792 24797322

[B94] Wiener-Megnazi Z. Vardi L. Lissak A. Shnizer S. Reznick A. Z. Ishai D. (2004). Oxidative stress indices in follicular fluid as measured by the thermochemiluminescence assay correlate with outcome parameters in *in vitro* fertilization. Fertil. Steril. 82 (3), 1171–1176. 10.1016/j.fertnstert.2004.06.013 15474091

[B95] Xu J. Bishop C. V. Lawson M. S. Park B. S. Xu F. (2016). Anti-Mullerian hormone promotes pre-antral follicle growth, but inhibits antral follicle maturation and dominant follicle selection in primates. Hum. Reprod. 31, 1522–1530. 10.1093/humrep/dew100 27165618 PMC4901882

[B96] Yen G. C. Chen Y. C. Chang W. T. Hsu C. L. (2011). Effects of polyphenolic compounds on tumor necrosis factor-α (TNF-α)-induced changes of adipokines and oxidative stress in 3T3-L1 adipocytes. J. Agric. Food Chem. 59, 546–551. 10.1021/jf1036992 21186817

[B97] Zhang F. Wang H. Wu Q. Lu Y. Nie J. Xie X. (2013a). Resveratrol protects cortical neurons against microglia-mediated neuroinflammation. Phytother. Res. 27, 344–349. 10.1002/ptr.4734 22585561

[B98] Zhang F. Wang Y.-Y. Yang J. Lu Y.-F. Liu J. Shi J.-S. (2013b). Tetrahydroxystilbene glucoside attenuates neuroinflammation through the inhibition of microglia activation. Oxid. Med. Cell. Longev. 2013, 680545. 10.1155/2013/680545 24349614 PMC3848273

[B99] Zhang L.-X. Li C.-X. Kakar M. U. Khan M. S. Wu P.-F. Amir R. M. (2021). Resveratrol (rv): A pharmacological review and call for further research. Biomed. Pharmacother. 143, 112164. 10.1016/j.biopha.2021.112164 34649335

[B100] Zhao J. Yan Y. Huang X. Li Y. (2020). Do the children born after assisted reproductive technology have an increased risk of birth defects? A systematic review and meta-analysis. J. Matern. Fetal. Neonatal Med. 33, 322–333. 10.1080/14767058.2018.1488168 30189770

[B101] Zhu X. Liu Q. Wang M. Liang M. Yang X. Xu X. (2011). Activation of Sirt1 by resveratrol inhibits TNF-α induced inflammation in fibroblasts. PLoS One 6, e27081. 10.1371/journal.pone.0027081 22069489 PMC3206084

[B102] Zwerschke W. Mazurek S. Stöckl P. Hütter E. Eigenbrodt E. Jansen-Dürr P. (2003). Metabolic analysis of senescent human fibroblasts reveals a role for AMP in cellular senescence. Biochem. J. 376, 403–411. 10.1042/BJ20030816 12943534 PMC1223775

