# Supplementary material for: 2,3,5,4′-Tetrahydroxystilbene-2-O-β-D-Glucoside improves female ovarian aging
Source: Front Cell Dev Biol. 2022 Aug 30;10:862045. doi: 10.3389/fcell.2022.862045 (PMC9469098; doi:10.3389/fcell.2022.862045)
Supplement: Supplementary file 1 [file DataSheet1.pdf]

Supplementary Material

MATERIALS AND METHODS

In vitro maturation (IVM)

Oocytes were collected from a group of young female mice (mice aged 8 to 36 weeks) and a group of aged female mice (mice aged 24 to 40 weeks) after superovulation. Oocytes were separated from surrounding cumulus cells using 0.3 mg/ml hyaluronidase (Sigma, St. Louis, MO, USA) to identify MII oocytes. After removing cumulus-corona cells, only the MII oocytes that have extruded the polar body (PB) were collected. PB and sibling oocytes were separated to collect MII oocytes that had extruded intact PB of normal size. Each MII oocyte was briefly treated with Tyrode's solution (Sigma) to remove its zona pellucida for facilitating oocyte maturation (Ubaldi and Rienzi, 2008;Velásquez et al., 2013). Oocyte then incubated with freshly prepared 7% ethanol for 5 minutes in M2 medium and after washing incubated in mature medium containing 10 μg/mL follicle-stimulating hormone (FSH), 10 μg/mL luteinizing hormone (LH), 10% fetal bovine serum, and incubated at 37℃ and 5% CO2. Activated oocytes were observed after 20 h. Oocytes further developed to the second polar body and mature oocytes within 2–3 days. The numbers of the polar body and mature oocyte were counted

Supplementary Figures

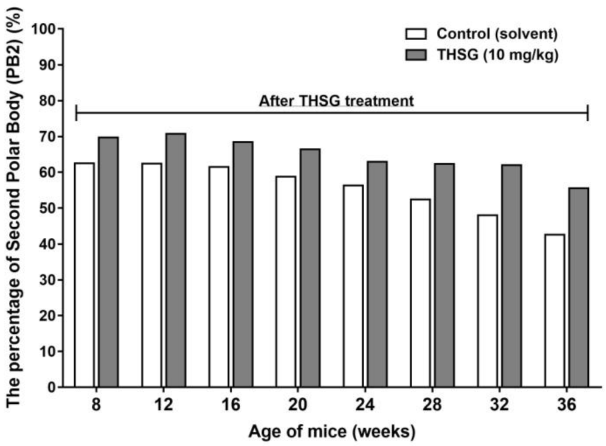

Supplementary Figure S1. The percentage of secondary polar body (PB2) in young mice groups. Female C57BL/6J mice (4 week-old) were received 10 mg/kg THSG (in 50% ethanol; experiment group) or solvent (50% ethanol; control group) for 32 weeks via gavage feeding. Collected oocytes were activated by treating with 7% ethanol. The percentage of secondary polar body (PB2) formation was recorded.

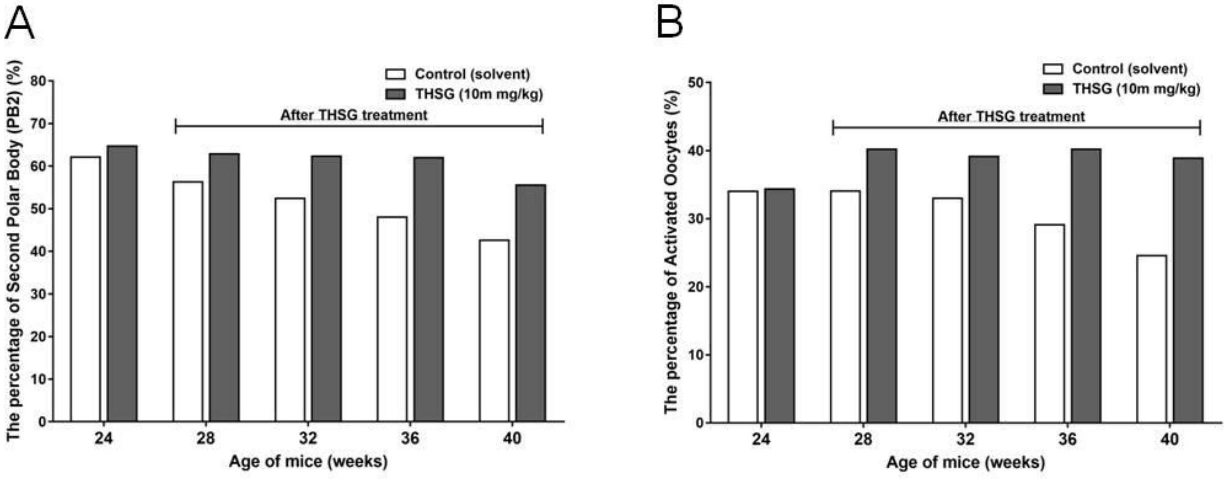

Supplementary Figure S2. The percentage of secondary polar body (PB2) and activated oocytes in aged mice groups. Female C57BL/6J mice (24 week-old) were received 10 mg/kg THSG (in 50% ethanol; experiment group) or solvent (50% ethanol; control group) for 16 weeks via gavage feeding. (A) Collected oocytes were activated by treating with 7% ethanol. The percentage of secondary polar body (PB2) formation was recorded. (B) The activated oocytes were collected after *in vitro* maturation (IVM).

REFERENCES

Ubaldi, F., and Rienzi, L. (2008). Morphological Selection of Gametes. *Placenta* 29**,** 115-120.

Velásquez, A., Manríquez, J., Castro, F., and Rodríguez-Alvarez, L. (2013). Effect of zona pellucida removal on early development of in vitro produced bovine embryos. *Archivos de medicina veterinaria* 45**,** 7-15.
